# Self-Fitting Versus Professional-Fitting Hearing Aids: A Systematic Review and Meta-Analysis of Randomized Controlled Trials

**DOI:** 10.3390/audiolres16050139

**Published:** 2026-09-19

**Authors:** Ebraheem Albazee, Hajar Alismail, Noof Albannai, Yaqoub Yousef Alenezi, Abdullah M. Alharran, Maya Yassir H. Ibrahim, Durar Ahmed Aljishi, Ahmed Abu-Zaid

**Affiliations:** 1 Otorhinolaryngology-Head and Neck Surgery, Kuwait Institute for Medical Specializations (KIMS), Kuwait City 13018, Kuwait; 2 Department of Otolaryngology-Head and Neck Surgery, Al-Jahra Hospital, Al-Jahra 03200, Kuwait; 3 Kuwait Institute for Medical Specializations (KIMS), Kuwait City 13018, Kuwait; hajaralismaill@gmail.com; 4 School of Medicine, Trinity College Dublin, D02 PN40 Dublin, Ireland; albannainouf@gmail.com; 5 College of Medicine and Medical Sciences, Arabian Gulf University, Manama 26671, Bahrain; yaqoub2002al@gmail.com (Y.Y.A.); dr_alharran@outlook.com (A.M.A.); 6 College of Medicine, Alfaisal University, Riyadh 11533, Saudi Arabia; myibrahim@alfaisal.edu (M.Y.H.I.); daljishi@alfaisal.edu (D.A.A.)

**Keywords:** over the counter, self-fitting, audiologist fitting, hearing loss, speech-in-noise test, APHAB, systematic review, meta-analysis

## Abstract

**Background:** Adult hearing loss is common, yet hearing-aid uptake remains inadequate due to systemic and financial barriers in traditional audiologist-based fitting models. Over-the-counter (OTC) and self-fitting strategies have emerged to improve accessibility, allowing users to select and adjust their devices independently. However, concerns remain that reducing professional involvement may compromise electroacoustic accuracy, speech-in-noise outcomes, and overall patient satisfaction. **Methods:** A comprehensive literature search across PubMed, Web of Science, CENTRAL, Scopus, and Embase was conducted up to 1 June 2026. Parallel-group and crossover randomized controlled trials (RCTs) comparing self-fitting with professional-fitting were included. The primary outcome was patient-reported hearing benefit. Standardized mean differences (SMDs) and risk ratios (RRs) were pooled using a random-effects model. **Results:** Seven RCTs involving 1092 participants were included. The analysis revealed no statistically significant difference between self-fitting and professional-fitting strategies regarding patient-reported hearing benefit (SMD: 0.01, 95% CI [−0.44, 0.46], *p* = 0.97, I^2^ = 89.2%, very low certainty of evidence). Similarly, no significant differences were observed across secondary outcomes, including speech-in-noise performance (SMD: 0.11, 95% CI [−0.25, 0.47]; *p* = 0.55), hearing loss (*p* = 0.12), hearing-aid satisfaction (*p* = 0.31), device adherence (*p* = 0.65), or the need for follow-up adjustment (RR: 0.46, 95% CI [0.12, 1.71]; *p* = 0.25). Safety outcomes were sparse and did not demonstrate a statistically significant difference between the fitting approaches; however, the evidence was imprecise and inconclusive. **Conclusions:** Self-fitting hearing aids showed no clear differences from professional best-practice fitting across several patient-reported and functional outcomes in adults with uncomplicated mild-to-moderate hearing loss. However, the certainty of evidence was very low because of risk of bias, substantial heterogeneity, imprecision, and the small number of trials. These findings are therefore hypothesis-generating and suggest that well-designed self-fitting pathways may have potential as an accessible alternative for selected users, but larger, rigorous, and longer-term trials are needed before broader clinical implementation can be recommended.

## 1. Introduction

Adult hearing loss is a common condition associated with impaired communication, social isolation, and decreased quality of life [1,2]. Other consequences of untreated hearing loss include accelerated cognitive and psychosocial decline [3,4]. Hearing aids remain the most effective intervention for patients with mild-to-moderate sensorineural hearing loss (SNHL) [5]. However, despite the well-established clinical benefits, the overall hearing-aid uptake remains inadequate, and a large portion of the affected population remains without adequate care [6,7].

Traditional hearing-aid delivery typically consists of extensive professional evaluation, individualized device selection, acoustic fitting, real-ear verification, and follow-up adjustments [8]. This professional model can provide optimized electroacoustic amplification and patient-centered fitting. Still, it is often limited by high out-of-pocket costs, limited access to audiology services, travel burdens, and time constraints [9]. Accordingly, these systemic and financial challenges limit device uptake, especially among adults with mild-to-moderate hearing loss who may delay or avoid seeking professional hearing care [10].

To address these challenges, over-the-counter (OTC) and self-fitting strategies have been proposed as alternative service models. These models can allow users to independently select, physically fit, and adjust their devices through preset programs, smartphone apps, or automated self-fitting algorithms [11,12]. However, while these models can reduce the need for in-person professional care and extend rehabilitation to underserved populations, there remain some concerns [13]. Removing or reducing professional involvement may compromise electroacoustic fitting accuracy, objective aided benefits, speech-in-noise outcomes, overall patient satisfaction, and long-term device adherence [14].

Recent randomized controlled trials (RCTs) have compared self-fitting, OTC-style hearing-aid models with audiologist-fitted or best-practice clinical models [15,16,17,18,19,20,21,22]. However, these trials vary in their methodological design, self-fitting approaches, follow-up durations, and whether the professional fitting arm reflects clinical best practice. Accordingly, the current evidence on the comparative efficacy of self-fitting versus professional-fitting devices remains uncertain. Therefore, this systematic review and meta-analysis aim to synthesize RCTs evaluating self-fitting versus professional-fitting hearing aids in adults with mild-to-moderate hearing loss using OTC-style hearing-aid models.

## 2. Methodology

### 2.1. Review Protocol and Reporting Style

Ethical approval was not required as this meta-analysis relied exclusively on previously published studies. The study design and reporting adhered to the Preferred Reporting Items for Systematic Reviews and Meta-Analyses (PRISMA) guidelines [23]. The review protocol was registered in the PROSPERO database (registration ID: CRD420261445071).

### 2.2. Information Sources and Search Strategy

A comprehensive systematic literature search was conducted across five electronic databases, including PubMed, Scopus, the Cochrane Central Register of Controlled Trials (CENTRAL), Embase, and Web of Science (WoS), from inception to 1 June 2026. The search strategy combined terms related to adult hearing loss, hearing aids, OTC, self-fitting delivery models, and professional or audiologist-based fitting. The exact search strategies that were used for each database are depicted in Table S1. No restrictions were imposed regarding language, country of origin, or publication status.

To enhance the completeness of the systematic search, the reference lists of all included studies were manually reviewed, and trial registries (i.e., ClinicalTrials.gov and ICTRP) were searched. Additional sources (i.e., ResearchGate and medRxiv) were also examined to identify potentially eligible unpublished studies. When required, corresponding authors were contacted to obtain missing information or to clarify study details.

### 2.3. Eligibility Criteria

We included parallel-group and crossover RCTs according to the following PICO framework:

- **Population:** Adults aged 18 years or older with perceived or confirmed mild-to-moderate hearing loss who were candidates for hearing-aid use.
- **Intervention:** OTC-style fitting strategies, including self-fitting, app-based self-fitting, preset-based self-fitting, consumer-decides, person-fit, or participant-directed hearing-aid fitting models in which participants independently selected, fitted, adjusted, or programmed the hearing aid without full individualized audiologist best-practice fitting.
- **Comparator:** Audiologist professional-fit or best-practice hearing-aid fitting, including hearing-aid selection, programming, counseling, verification, or adjustment delivered by a hearing-care professional.
- **Outcomes:** The primary outcome was self-reported hearing-aid benefit, measured using the abbreviated profile of hearing aid benefit (APHAB), PHAB, Glasgow hearing aid benefit profile (GHABP), or an equivalent score. Secondary outcomes included speech-in-noise performance, hearing loss, hearing-aid satisfaction, hearing-aid use adherence, device retention/willingness to keep hearing aids, need for follow-up adjustment, and adverse events.

We excluded non-randomized studies, observational studies, conference abstracts, protocols, reviews, editorials, and studies without a directly eligible comparator. For studies with multiple relevant intervention arms, eligible OTC/self-fit arms were combined when clinically appropriate to generate a single comparison against the audiologist-fit group. Specifically, self-fitting or consumer-directed arms were classified under the OTC/self-fit group, whereas professional or best-practice fitting arms were classified under the audiologist-fit group.

### 2.4. Study Selection Process

Records were managed and screened using an electronic platform. Two investigators (HA and NA) independently screened studies in a two-stage process, beginning with title and abstract screening, followed by full-text assessment of potentially eligible articles. Disagreements were resolved through consultation with a third reviewer (EA). The same approach was applied during risk of bias assessment (YYA and AMA) and statistical analysis (EA and AMA). When studies reported multiple eligible outcome measures or follow-up assessments, the measure and time point most directly corresponding to the prespecified outcome were selected, while avoiding duplicate inclusion of participants from the same trial. Where multiple publications reported the same trial or cohort, including extension or follow-up reports, they were treated as a single study and data were collated accordingly.

### 2.5. Data Extraction

A standardized Excel extraction form was developed and pilot-tested using a subset of eligible studies. Extracted data were grouped into three categories. First, study characteristics included author, year, country, study design, trial setting, recruitment period, total sample size, follow-up duration, hearing-aid device type, fitting approach, comparator fitting protocol, and key details of the intervention and professional fitting procedures. Second, baseline participant characteristics included age, sex, prior hearing-aid experience, proportion of hearing-aid-naïve participants, hearing-loss severity, pure-tone average definition and values, baseline speech-in-noise or word-recognition performance, and baseline patient-reported hearing difficulty. Third, outcome data included all primary and secondary outcomes described above.

Dichotomous outcomes were extracted as the number of events and the total number of randomized or analyzed participants. Continuous outcomes were extracted as means and standard deviations (SDs). When means and SDs were not directly reported, standard conversion methods were applied. Outcomes reported as medians and interquartile ranges were converted to means and SDs using the methods of Wan et al. [24]. When studies reported 95% confidence intervals around a mean without SDs, SDs were calculated algebraically from the confidence interval limits according to the Cochrane Handbook [25]. For change-from-baseline outcomes in which the SD of change was not reported, the SD was imputed using baseline and final SDs with a conservative correlation coefficient of 0.5, following Follmann et al. [26]. When two eligible intervention arms required combination into a single OTC/self-fit group, pooled sample sizes, means, and SDs were calculated using standard combined-group formulae.

### 2.6. Risk of Bias and Certainty of Evidence

The risk of bias for each included randomized trial was assessed using the revised Cochrane Risk of Bias 2 (RoB-2) tool [27]. The assessment considered bias arising from the randomization process, deviations from intended interventions, missing outcome data, measurement of the outcome, and selection of the reported result. For crossover trials, additional considerations relevant to period, carryover, and allocation sequence were considered where applicable. Disagreements were resolved through discussion and consensus.

The certainty of evidence for each major outcome was evaluated using the Grading of Recommendations Assessment, Development and Evaluation (GRADE) framework [28,29]. The certainty of evidence was rated across the domains of risk of bias, inconsistency, indirectness, imprecision, and publication bias. All judgments were justified and documented.

### 2.7. Meta-Analysis and Data Synthesis

All statistical analyses were performed using Stata/SE software, version 19 (StataCorp, College Station, TX, USA). Dichotomous outcomes were analyzed using risk ratios (RRs), while continuous outcomes were analyzed using standardized mean differences (SMDs) as the included studies used different scales; all effect estimates were reported with 95% confidence intervals (CIs). Because the included studies differed in hearing-aid technology, fitting algorithms, degree of professional involvement, and follow-up duration, clinical heterogeneity was anticipated a priori. Random-effects meta-analysis was therefore selected as the primary approach. Leave-one-out sensitivity analyses and Galbraith plots were used to explore the influence of individual studies on statistical heterogeneity; these analyses were interpreted as exploratory and were not used to justify exclusion of studies solely on the basis of statistical significance. For dichotomous outcomes with zero events in one arm, a continuity correction of 0.5 was applied. Statistical heterogeneity was assessed using the Chi-squared test and quantified using the I^2^ statistic. Significant heterogeneity was defined as a chi-squared *p*-value < 0.01 or an I^2^ value > 50%. In case of significant heterogeneity, a leave-one-out sensitivity analysis was conducted to explore the source of heterogeneity. Publication bias was not assessed because all meta-analyzed outcomes included fewer than 10 trials [30].

For crossover trials, outcome data were extracted according to the reporting format of the original studies. Because participant-level paired data and within-participant correlation coefficients were not available in the published reports, the available condition-level summary statistics were used in the meta-analysis without introducing an assumed correlation coefficient. This limitation was considered when interpreting the certainty of the evidence. Given the small number of eligible RCTs, crossover trials were retained in the primary analysis rather than excluded in a separate sensitivity analysis, as excluding them would further reduce the already limited evidence base.

## 3. Results

### 3.1. Summary of the Systematic Review Search

**Figure 1:**
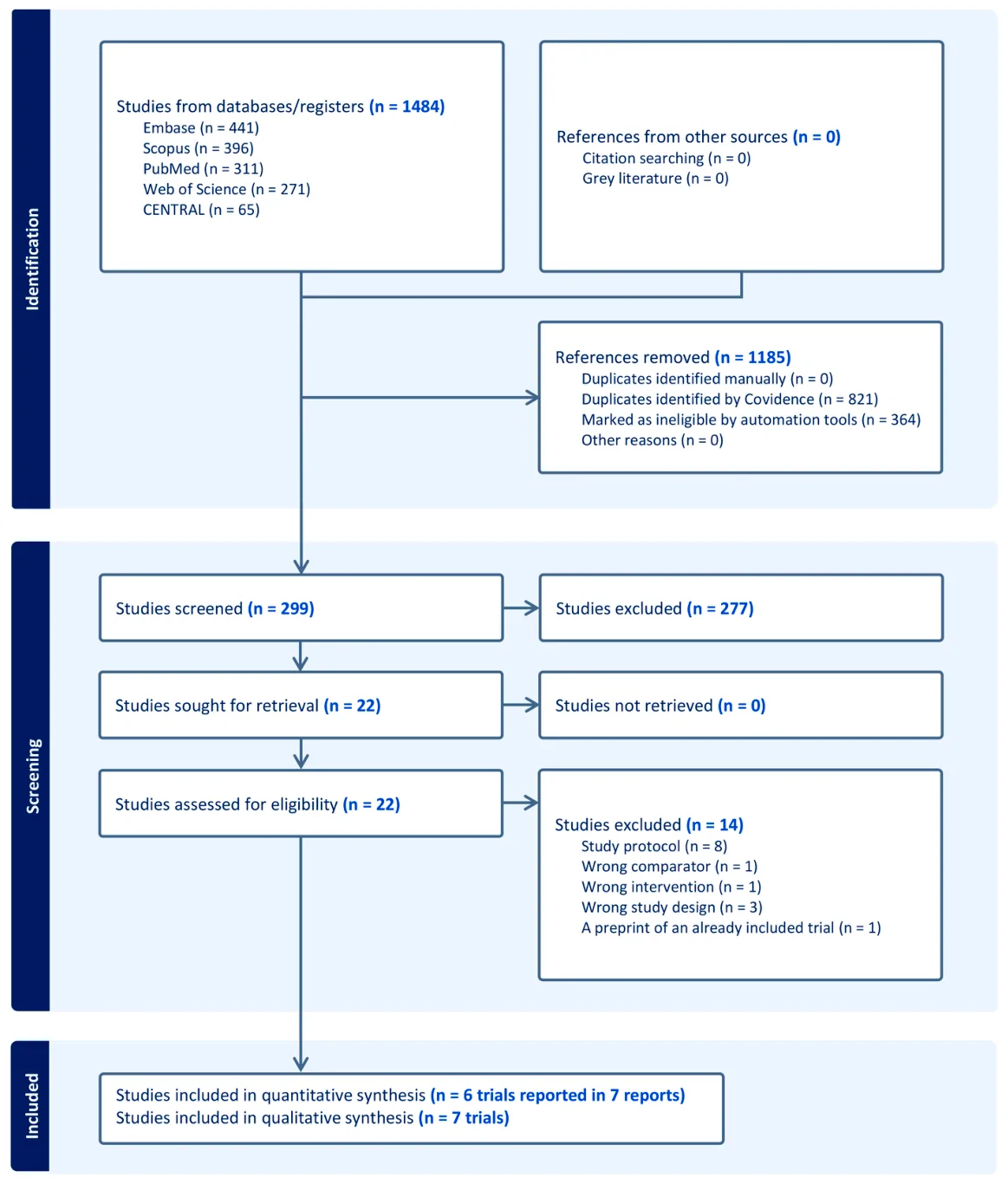
PRISMA flow chart of the study screening and selection process.

A total of 1484 records were retrieved from the initial literature search across five databases. Before screening, 1185 references were removed, including 821 duplicates identified by Covidence and 364 records marked as ineligible by Covidence automation tools. The remaining 299 records were screened, of which 277 were excluded after failing to meet the inclusion criteria. Twenty-two full-text reports were assessed for eligibility, and 14 were excluded for various reasons (Table S2). Finally, six trials reported across seven reports were included in the quantitative and qualitative synthesis [16,17,18,19,20,21,22], while one additional trial was included in the qualitative synthesis only [15] (Figure 1). Multiple publications arising from the same randomized cohort were treated as a single trial for purposes of participant counting and meta-analysis; specifically, De Sousa et al. 2023 [20] and De Sousa et al. 2024 [19] represent reports from the same randomized cohort and were not counted as independent trials.

### 3.2. Summary of Study Characteristics and Baseline Participant

Seven RCTs reported across eight articles, involving 1092 participants, were included in this review [15,16,17,18,19,20,21,22]. All were conducted in the United States [15,16,17,18,21,22], except for one trial from South Africa [19,20]. All trials enrolled adults with predominantly mild-to-moderate hearing loss. Wu et al. randomized 295 participants, of whom 245 completed follow-up. After excluding the OTC+ arms and combining technology levels within the OTC and AUD groups, 165 participants contributed to the meta-analysis (OTC, n = 82; AUD, n = 83). A key characteristic of the included evidence was variation in the OTC or self-fitting delivery model, ranging from commercially available self-fitting OTC devices and app-based fitting algorithms to simulated OTC models based on preset selection or consumer-directed fitting. Comparator arms generally involved audiologist-fitted or conventional best-practice care, commonly incorporating audiometric thresholds, NAL-NL2 targets, real-ear verification, counseling, and follow-up support. Follow-up duration ranged from short field-use periods of 2 weeks to longer assessments extending up to 8 months. Detailed study designs and baseline participant characteristics are summarized in Table 1 and Table 2, respectively. Finally, detailed descriptions of self-fitting and best-practice-fitting strategies across the included trials are provided in Tables S3 and S4, respectively.

### 3.3. Risk of Bias and Certainty of Evidence

**Figure 2:**
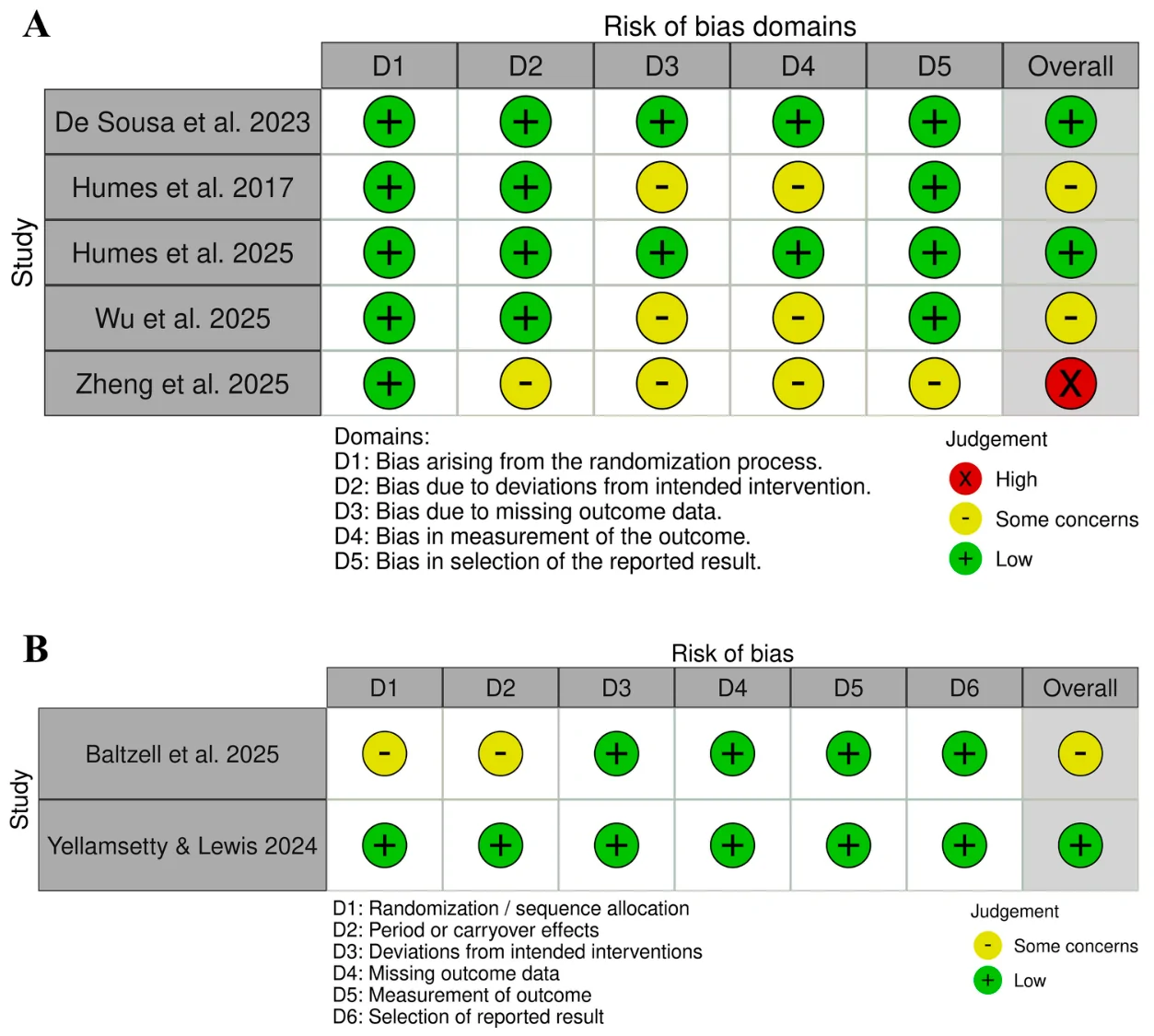
Quality assessment of risk of bias in the included randomized trials. Panel (**A**) presents the risk-of-bias judgments for parallel-group randomized trials using the RoB 2 tool [16,17,18,20,22], and Panel (**B**) presents the risk-of-bias judgments for randomized crossover trials [15,21]. Green indicates low risk, yellow indicates some concerns, and red indicates high risk of bias.

For parallel-group RCTs, two trials were judged to have a low overall risk of bias [17,20], while another two raised some concerns [16,18], and Zheng et al. showed a high overall risk of bias [22] (Figure 2A). The main drivers of concern ratings were bias due to missing outcome data and bias in the measurement of outcomes. For randomized crossover trials, Yellamsetty and Lewis [15] showed a low overall risk of bias, whereas Baltzell et al. [21] raised some concerns (Figure 2B). Additionally, the detailed GRADE certainty of evidence assessment is outlined in Table 3.

### 3.4. Primary Outcome: Patient-Reported Hearing Benefit

**Figure 3:**
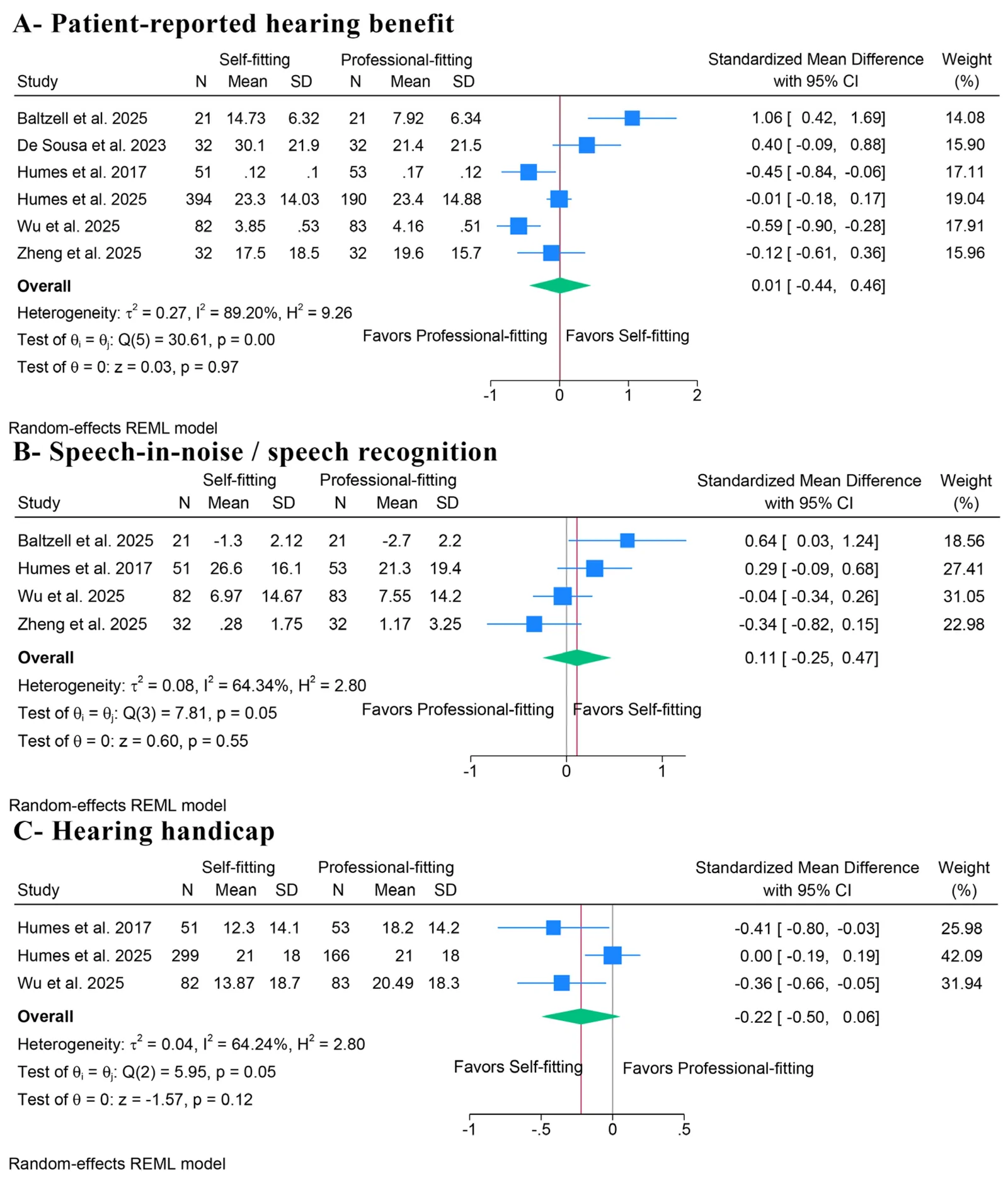
Forest plots comparing self-fitting and best practice-fitting for primary and secondary effectiveness outcomes. The forest plots display standardized mean differences for (**A**) patient-reported hearing benefit, (**B**) speech-in-noise/speech recognition, and (**C**) hearing loss [16,17,18,20,21,22].

There was no significant difference between the two groups (SMD: 0.01, 95% CI [−0.44, 0.46], *p* = 0.97, I^2^ = 89.2%) (Figure 3A). Leave-one-out sensitivity analysis showed that the pooled effect remained statistically non-significant after omitting each study (Figure S1). However, the Galbraith plot suggested important between-study inconsistency, with Baltzell et al. and Wu et al. appearing as potential outliers (Figure S2).

## 4. Secondary Outcomes

### 4.1. Speech-in-Noise/Speech Recognition

There was no significant difference between the two groups (SMD: 0.11, 95% CI [−0.25, 0.47], *p* = 0.55, I^2^ = 64.34%) (Figure 3B). Leave-one-out sensitivity analysis showed that the pooled effect remained statistically non-significant after omitting each study (Figure S3). The Galbraith plot did not identify any clear outliers, although the individual effects varied in direction, which explains the moderate heterogeneity (Figure S4).

### 4.2. Hearing Loss

There was no significant difference between the two groups (SMD: −0.22, 95% CI [−0.50, 0.06], *p* = 0.12, I^2^ = 64.24%) (Figure 3C). Sensitivity analysis suggested that this result was partly influenced by Humes et al. 2025 [17], as after omitting this study, the pooled estimate became statistically significant (SMD: −0.38, 95% CI [−0.62, −0.14], *p* = 0.002), favoring self-fitting (Figure S5). The Galbraith plot did not show a clear outlier (Figure S6).

### 4.3. Hearing-Aid Satisfaction

**Figure 4:**
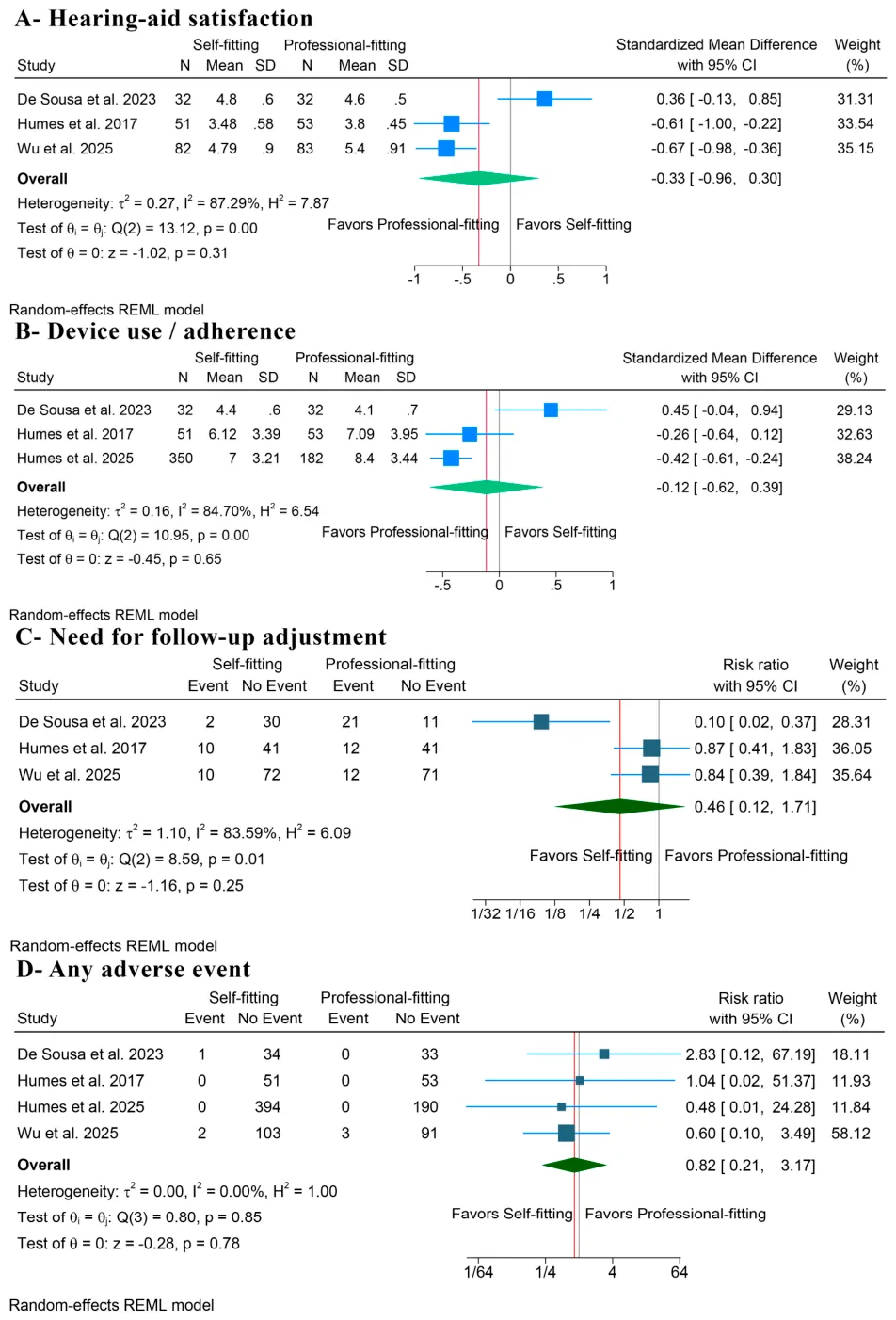
Forest plots comparing self-fitting and best practice-fitting for use-related, patient-reported, and safety outcomes. The forest plots display standardized mean differences for (**A**) hearing-aid satisfaction and (**B**) device use/adherence, and risk ratios for (**C**) need for follow-up adjustment and (**D**) any adverse event [16,17,18,20].

There was no significant difference between the two groups (SMD: −0.33, 95% CI [−0.96, 0.30], *p* = 0.31, I^2^ = 87.29%) (Figure 4A). Leave-one-out sensitivity analysis indicated that this finding was sensitive to the exclusion of De Sousa et al. 2023 [20], as omitting this study made the pooled estimate statistically significant (SMD: −0.65, 95% CI [−0.89, −0.40], *p* < 0.001), favoring best-practice fitting (Figure S7). The Galbraith plot contributed to this instability, showing De Sousa et al. 2023 [20] and Wu et al. 2025 [16] as the main contributors to heterogeneity (Figure S8).

### 4.4. Device Use/Adherence

There was no significant difference between the two groups (SMD: −0.12, 95% CI [−0.62, 0.39], *p* = 0.65, I^2^ = 84.70%) (Figure 4B). Sensitivity analysis showed that the pooled estimate became statistically significant after omitting De Sousa et al. 2023 [20] (SMD: −0.40, 95% CI [−0.56, −0.23], *p* < 0.001), favoring best-practice fitting (Figure S9). The Galbraith plot identified De Sousa et al. 2023 [20] and Humes et al. 2025 [17] as the principal contributors to heterogeneity (Figure S10).

### 4.5. Need for Follow-Up Adjustment

There was no significant difference between the two groups (RR: 0.46, 95% CI [0.12, 1.71], *p* = 0.25, I^2^ = 83.59%) (Figure 4C). Leave-one-out sensitivity analysis showed that the result remained statistically non-significant after omitting each study (Figure S11). The Galbraith plot suggested that De Sousa et al. 2023 [20] was the main contributor to heterogeneity (Figure S12).

### 4.6. Any Adverse Event

There was no statistically significant difference between the self-fitting and best-practice fitting groups in the incidence of any adverse event (RR: 0.82, 95% CI [0.21, 3.17], *p* = 0.78, I^2^ = 0%). However, the evidence was sparse and imprecise, and the wide confidence interval does not exclude clinically important differences in either direction (Figure 4D).

## 5. Discussion

Across the seven included RCTs and 1092 patients, there was no statistically significant difference between self-fitting and best-practice fitting for patient-reported hearing benefit, speech-in-noise/speech-recognition performance, hearing loss, hearing-aid satisfaction, device use/adherence, need for follow-up adjustment, or adverse events. These findings may suggest that, under trial conditions and in selected adult patients, self-fitting models can achieve outcomes similar to those of professional-fitting models. However, the certainty of the evidence was very low across outcomes due to risk-of-bias concerns, considerable heterogeneity, imprecision, and the small number of trials included.

The absence of statistically significant differences across several outcomes should not be interpreted as evidence that professional fitting is unnecessary or that self-fitting is equivalent to professional fitting. Given the very low certainty of evidence and the limitations of the available trials, the findings only indicate that a clear advantage of professional fitting was not demonstrated in the populations and conditions studied. This is consistent with the rationale for the OTC hearing-aid introduction: to improve access and affordability by allowing adults with perceived mild-to-moderate hearing loss to obtain hearing aids without a mandatory medical examination, prescription, or audiologist-fitting adjustment [31,32]. Also, the FDA has recently granted authorization for an OTC hearing aid software that enables personalized amplification based on the user’s hearing profile, which contributes to the ongoing regulatory and technological transition toward the independent or semi-independent fitting of hearing devices [33].

Within the included evidence, De Sousa et al. showed comparable short-term outcomes between a self-fitting OTC device and audiologist fitting [20]. Baltzell et al. found that a self-fitted OTC intervention was not inferior to a clinician-fitted intervention for self-reported benefit and speech-in-noise outcomes [21], and Zheng et al. reported no clear patient-reported advantage of professional fitting over self-fitting for an OTC device [22]. Related self-fitting and OTC studies also suggest that autonomous fitting methods can provide a clinically meaningful benefit when device design, fitting algorithms, and user guidance are adequate [34]. Thus, these findings raise the possibility that optimized self-fitting strategies could provide an alternative to routine professional fitting for a subset of appropriately selected users. However, given the very low certainty of evidence, this possibility should be considered hypothesis-generating rather than evidence of established clinical equivalence. In that model, audiologists remain essential. Still, their role shifts from an obligatory first-line fitting for all users toward diagnostic triage, counseling, troubleshooting, and escalation for users who fail or are unsuitable for self-fitting.

Given the small number of eligible trials and the limited number of studies contributing to several outcomes, further subgroup analyses by fitting model, device technology, or follow-up duration were not considered sufficiently reliable or informative. Accordingly, the pooled estimates should be interpreted in the context of substantial clinical and methodological heterogeneity, and the findings should not be generalized across all self-fitting technologies or service-delivery models. Baltzell et al. evaluated an app-guided self-fitting device in a crossover design [21]. De Sousa et al. tested the Lexie Lumen self-fitting hearing aid against an audiologist-fitted version of the same device, with long-term outcomes reported separately [19,20]. Humes et al. evaluated a consumer-decides model and later compared self-fit/person-fit methods with audiologist best-practice fitting in a large multisite trial [17,18]. Wu et al. compared different service models and technology levels [16], while Zheng et al. compared self-fit and professional-fit modes of an OTC device [22]. Therefore, this may indicate that, for some adults with uncomplicated mild-to-moderate hearing loss, well-designed self-fitting pathways can approximate the patient-perceived benefit of professional fitting. However, the high heterogeneity suggests that this replacement potential is likely affected by the self-fitting strategy, and an optimal method remains unknown.

Moreover, the lack of a significant difference in speech-in-noise or speech-recognition outcomes also supports the possibility that professional fitting may not always be required to achieve routine functional benefit. Speech understanding in noise is one of the most clinically relevant problems for adults with sensorineural hearing loss and is not fully captured by simple audibility measures [35,36]. Although professional fitting would be expected to improve target matching through audiometric programming, real-ear verification, fine-tuning, and counseling, this did not translate into a consistent pooled advantage. These findings suggest that a clear pooled advantage of professional fitting was not demonstrated for the speech outcomes assessed; however, they do not establish that automated or user-guided fitting provides equivalent speech-in-noise or speech-recognition benefits. However, laboratory speech tests may not fully reflect real-world listening conditions, where competing talkers, fatigue, cognitive load, and changing environments can affect communication success [35,37]. Future trials should therefore test whether optimized self-fitting strategies remain comparable to professional fitting using real-world speech understanding, listening effort, ecological momentary assessment, and daily-use data.

Importantly, the applicability of these findings to routine real-world hearing-aid use remains uncertain. Most included trials evaluated relatively short-term outcomes in selected adults with uncomplicated mild-to-moderate hearing loss and under trial conditions that may not fully represent the complexity of everyday listening environments. Important real-world outcomes, including sustained long-term adherence, listening effort in complex acoustic environments, device management, troubleshooting, and help-seeking behavior, remain insufficiently studied for self-fitting pathways. Therefore, the absence of significant differences in the available trial-based outcomes should not be interpreted as evidence that self-fitting and professional fitting provide equivalent long-term real-world experiences. Future research should prioritize pragmatic trials embedded within routine clinical and community settings, with longer follow-up and ecologically relevant measures such as ecological momentary assessment, objectively measured daily device use, listening effort, device-management burden, and patterns of help-seeking and professional support.

Furthermore, findings on satisfaction and device use/adherence can support the idea that hearing outcomes are shaped by implementation rather than by professional involvement alone. Hearing-aid satisfaction depends on perceived sound quality, physical comfort, ease of use, expectations, cosmetic acceptance, cost, support availability, and perceived value [38,39]. Hence, the replacement potential of self-fitting devices depends less on simply removing audiologists from the pathway and more on whether self-fitting systems can reliably reproduce the components that drive real-world use. An optimized self-fitting pathway with clear instructions, adaptive fine-tuning, remote support, and easy return or escalation options may be more clinically relevant.

Supporting this, the need for follow-up adjustment also did not differ significantly. In professionally fitted pathways, follow-up may be actively scheduled or encouraged as part of best-practice care [40]. In contrast, in self-fitting pathways, adjustment may depend on whether users recognize a problem, know how to seek help, and have access to support. Thus, a lower rate of follow-up adjustment in a self-fitting arm may reflect fewer problems. Still, it may also reflect reduced access, lower expectations, or unmet needs that remain unrecognized. Conversely, a higher rate in a professional arm may reflect more proactive care rather than poorer initial fitting [40,41]. Accordingly, if self-fitting is to replace routine professional fitting for selected users, future studies should distinguish between patient-requested adjustment, clinician-recommended adjustment, successful troubleshooting, unmet adjustment needs, and device discontinuation, because these represent different aspects of service quality.

The temporal and technological evolution of hearing-aid devices also has implications for the generalizability of these findings. Newer self-fitting algorithms, smartphone-based interfaces, automated personalization, and remote-support models may perform differently from earlier approaches included in this review. Conversely, improvements in professional fitting technologies and service delivery may also influence the magnitude of the difference between fitting pathways. Thus, the absence of a pooled difference should not be interpreted as evidence that self-fitting and professional fitting are equivalent across all current or future technologies. Future trials should use contemporary devices, clearly characterize the fitting algorithm and level of professional support, and report outcomes in a manner that permits comparison across technological generations and service models.

This review has several strengths. It focused on RCTs, reducing confounding compared with observational evidence. It included both parallel-group and crossover RCTs, assessed commercially available self-fitting OTC devices, and examined a broad set of clinically relevant outcomes. Risk of bias was assessed using RoB 2; certainty was judged using the GRADE framework; and sensitivity analyses were used to explore the influence of individual studies.

However, several limitations should also be acknowledged. First, the number of included trials was small, and most were conducted in the United States, limiting generalizability across geography and health systems. Second, several outcomes included only three or four trials, and publication bias could not be reliably assessed. Third, follow-up was short in many studies, limiting conclusions about long-term device retention, sustained use, and late adverse events. Fourth, some trials experienced attrition, missing outcome data, or concerns about the measurement and reporting of outcomes. Fifth, the self-fitting interventions varied extensively, ranging from app-based in situ algorithms to preset selection and consumer-decides approaches. At the same time, professional-fitting strategies also differed. Sixth, the outcome assessment scores differed across the included trials, and the use of SMD allowed pooling across instruments but may have affected the interpretation of the results. An additional methodological limitation is that two included trials used a crossover design, but participant-level paired data and within-participant correlation estimates were not available from the published reports. Consequently, the available condition-level summary data were used, which may not fully account for the statistical dependence inherent to crossover designs. This limitation contributes to uncertainty in the corresponding pooled estimates and was considered in the overall assessment of evidence certainty. Seventh, the selected nature of trial participants and predominantly short follow-up periods limit the generalizability of the findings to routine real-world use. Participants enrolled in RCTs may be more motivated, digitally capable, or clinically suitable for self-fitting than users encountered in routine clinical or community settings, who may have greater variation in hearing-loss characteristics, digital skills, support needs, and ability to manage hearing aids independently. Therefore, the findings may be applicable primarily to selected uncomplicated users and should not be generalized to all adults with hearing loss. An additional limitation is variation in professional contact and follow-up support across trials. These implementation details were inconsistently reported, precluding reliable quantitative analysis. Thus, observed differences may partly reflect variation in service support rather than fitting strategy alone. A further limitation is the temporal and technological evolution of self-fitting and OTC hearing aids across studies. The small number of eligible RCTs and substantial variation in devices, fitting algorithms, and service models precluded reliable subgroup or meta-regression analyses by publication year or technology generation. Because publication year is also an imperfect proxy for technological advancement, the pooled findings should not be interpreted as demonstrating stable equivalence across technological eras. Finally, given these limitations, the current certainty of the evidence remains very low, warranting careful interpretation.

For appropriately selected adults with uncomplicated mild-to-moderate hearing loss, self-fitting hearing aids may have potential as an access-oriented fitting pathway. However, the current evidence is insufficient to establish equivalence or non-inferiority to professional fitting and therefore does not support replacing routine professional fitting as a general practice. Professional care remains important for users who fail or are unsuitable for self-fitting, prefer clinician support, have more severe hearing loss, or have complex audiologic needs.

Accordingly, future research should move beyond asking only whether self-fitting works and should instead identify which self-fitting strategy can safely and consistently replace routine professional fitting, and for which patient subgroup. Large pragmatic non-inferiority or equivalence RCTs are needed to compare contemporary commercially available OTC/self-fitting devices with clearly defined audiologist best-practice fitting. Follow-up should extend to at least 6–12 months because early benefit does not necessarily predict sustained adherence. Future trials should also stratify participants by age, prior hearing-aid experience, degree of hearing loss, digital literacy, socioeconomic status, and cognitive status. Finally, future research can test optimized replacement strategies, including fully autonomous self-fitting, OTC plus remote support, OTC plus optional real-ear verification, OTC plus audiology triage, and stepped referral models. Such designs can determine whether professional fitting is best reserved for selected cases rather than routinely required for all users.

## 6. Conclusions

The available randomized evidence did not demonstrate clear differences between self-fitting hearing aids and best-practice professional fitting for patient-reported benefit, speech outcomes, hearing loss, satisfaction, adherence, follow-up adjustment, or adverse events, although the evidence for adverse events was sparse and imprecise. However, the certainty of evidence was very low, and substantial heterogeneity, risk of bias, imprecision, and the small number of trials limit confidence in these findings. Therefore, the results should be considered hypothesis-generating rather than evidence that self-fitting can broadly replace professional fitting. Self-fitting may have potential as an accessible option for selected adults with uncomplicated mild-to-moderate hearing loss; however, larger, rigorous, and longer-term trials across diverse clinical and service-delivery settings are needed to establish its effectiveness, safety, and appropriate role relative to professional care.

## Figures and Tables

**Table 1:** Summary characteristics of the included RCTs.

Study ID	Study Design	Country	Total Participants	Hearing Loss Definition/PTA	Prior Hearing-Aid Experience	Main Inclusion Criteria	Follow-Up Duration	Attrition/Loss to Follow-Up	Primary Outcome
Baltzell et al. 2025 [21]	Within-subject crossover RCT	USA	21	Four-frequency pure-tone average (PTA) at 05, 1, 2, and 4 kHz; at least one threshold > 20 dB HL with limits at specific frequencies	17/21 new users; 4/21 experienced users	Adults ≥ 18 years old, able to understand English, with mild-to-moderate hearing loss and normal middle ear function	2-week field-use period for each intervention condition	2 of the 23 eligible participants were lost to follow-up/did not complete	Self-reported benefit (APHAB) and objective speech-in-noise benefit (QuickSIN)
De Sousa et al. 2023 [20]	Parallel group RCT	South Africa	64	PTA at 05, 10, 20, and 40 kHz; excluded if thresholds < 20 dB HL at all frequencies or >80 dB HL at two or more frequencies	Mixed: AF 6/32 experienced; SF 11/32 experienced; remainder new users	Adults ≥ 18 years old with self-reported mild to moderate hearing loss and no history of outer/middle ear disease in the past 90 days	2 weeks and 6 weeks post-fitting	4 of the 68 randomized participants were excluded or lost to follow-up	Self-reported hearing aid benefit measured via the APHAB questionnaire
De Sousa et al. 2024 [19]	Long-term extension of De Sousa 2023 RCT	South Africa	44	PTA at 05, 10, 20, and 40 kHz, consistent with the original trial’s audiometric criteria	Mixed: AF 26 new/6 experienced; SF 21 new/10 experienced	Participants from the original 6-week non-inferiority trial who were not lost to follow-up	Approximately 8 months post-fitting	20 of the original 64 participants were lost to long-term follow-up due to nonresponse	Self-reported hearing aid benefit measured using the APHAB and the IOI-HA questionnaires
Humes et al. 2017 [18]	Parallel group RCT	USA	154	PTA (500, 1000, 2000 Hz) averaged ~26–28 dB HL; high-frequency PTA (1000, 2000, 4000 Hz) averaged ~38–39 dB HL	Required no prior hearing-aid experience	Adults 55–79 years old, native English speakers, MMSE-2 SV > 25, no prior HA experience, with symmetrical age-related hearing loss	6-week trial (Session 3) followed by an optional 4-week trial for CD and placebo groups	9 participants out of 163 withdrew after their initial session (154 analyzed)	Aided benefit measured by the 66-item Profile of Hearing Aid Benefit (PHAB)
Humes et al. 2025 [17]	Parallel group RCT	USA	584	Better-ear 4-frequency PTA (500, 1000, 2000, 4000 Hz) ≤ 50 dB HL	Required never used/tried hearing aids	Adults 50–79 years, no HA experience, English speakers, HHIE score > 4, MoCA score ≥ 23, able to pay $650	6 weeks and 6 months post-fitting	119 participants out of 584 withdrew or were lost to follow-up by the 6-month mark	Global score from the Profile of Hearing Aid Benefit (PHAB)
Wu et al. 2025 [16]	Two-site randomized factorial trial: 3 service models × 2 technology levels	USA	165	PTA (05, 1, 2, and 4 kHz) between 25 and 65 dB HL	Required no previous hearing-aid experience	Adults aged 55 to 85 years with bilateral sensorineural hearing loss, native English speakers, and no previous HA experience	6 to 7 weeks post-fitting	50 participants withdrew out of 295 randomized trials (245 completed)	Glasgow Hearing Aid Benefit Profile (GHABP) administered using ecological momentary assessment (EMA)
Yellamsetty and Lewis 2024 [15]	Two-site repeated-measures randomized crossover clinical trial	USA	40	WHO classification for 500–4000 Hz: slight (<25 dB HL), mild (26–40 dB HL), and moderate (41–55 dB HL)	Mostly experienced: 36/40 experienced users; 4/40 new users	Adults aged 18 to 80 with mild-to-moderate HL, computer/English proficiency, no significant neurological deficits, and with a preference for ≥1 year HA experience	2 weeks of field use per condition (4 weeks total)	0 reported lost to follow-up (all 40 participants evaluated)	Efficacy assessed via objective (REM), behavioral (QuickSIN), and subjective (APHAB) measures
Zheng et al. 2025 [22]	Parallel group RCT	USA	64	Air-conduction threshold between 25- and 60 dB HL with an air-bone gap < 15 dB; PTA assessed for 05, 1, 2, and 4 kHz	Mixed: pro-fit 11 nonusers/21 HA users; self-fit 12 nonusers/20 HA users	Native-English speakers ≥ 18 years old with mild-to-moderate sensorineural hearing loss and normal cognitive and middle ear functions	1 month	7 participants out of 71 randomized trials discontinued or were excluded	Aided benefit evaluated by the QuickSIN test, APHAB, SSQ12, and objective REAG/O measures

AF, audiologist fitting; APHAB, Abbreviated Profile of Hearing Aid Benefit; CD, consumer decides; dB, decibel; EMA, ecological momentary assessment; GHABP, Glasgow Hearing Aid Benefit Profile; HA, hearing aid; HHIE, Hearing Handicap Inventory for the Elderly; HL, hearing level; IOI-HA, International Outcome Inventory for Hearing Aids; kHz, kilohertz; MMSE-2 SV, Mini-Mental State Examination, Second Edition, Standard Version; MoCA, Montreal Cognitive Assessment; PHAB, Profile of Hearing Aid Benefit; PTA, pure-tone average; QuickSIN, Quick Speech-in-Noise test; RCT, randomized controlled trial; REAG/O, real-ear aided gain/output; REM, real-ear measurement; SF, self-fitted; SSQ12, Speech, Spatial and Qualities of Hearing Scale-12; USA, United States of America; WHO, World Health Organization.

**Table 2:** Baseline characteristics of the participants.

Study ID	Number of Patients in Each Group		Age (Years), Mean (SD)		Gender (Male), N. (%)		Prior Hearing-Aid Use, N. (%)		Hearing-Aid Naïve, N. (%)		PTA, Mean (SD)	
	Self-Fitting	Best Practice-Fitting	Self-Fitting	Best Practice-Fitting	Self-Fitting	Best Practice-Fitting	Self-Fitting	Best Practice-Fitting	Self-Fitting	Best Practice-Fitting	Self-Fitting	Best Practice-Fitting
Baltzell et al. 2025 [21]	21		66.2 (13.4)		13 (61.9%)		4 (19.1%)		17 (80.9%)		37.0 (10.1), PTA 0.5/1/2/4 kHz	
De Sousa et al. 2023 [20]	32	32	62.0 (13.1)	65.3 (14.9)	15 (46.9%)	18 (56.3%)	11 (34.4%)	6 (18.8%)	21 (65.6%)	26 (81.3%)	38.4 (11.7), PTA 0.5/1/2/4 kHz	41.8 (13.6), PTA 0.5/1/2/4 kHz
De Sousa et al. 2024 [19]	23	21	NR	NR	NR	NR	10/32 (31.3%), parent baseline	6/32 (18.8%), parent baseline	21/32 (65.6%), parent baseline	26/32 (81.3%), parent baseline	38.4 (11.7), parent baseline PTA 0.5/1/2/4 kHz	41.8 (13.6), parent baseline PTA 0.5/1/2/4 kHz
Humes et al. 2017 [18]	51	53	68.0 (6.2)	69.9 (5.6)	27 (49.1%)	28 (52.8%)	0	0	55/55 (100%)	53/53 (100%)	28.9 (8.6), bilateral PTA	28.7 (7.7), bilateral PTA
Humes et al. 2025 [17]	394	190	69.4 (6.4)	68.4 (6.9)	NR arm-specific; across fitting groups, males ranged 53.9–58.4%		0	0	394 (100%)	190 (100%)	Better-ear PTA4 32.0 (9.2), calculated; worse-ear PTA4 36.7 (9.7), calculated	Better-ear PTA4 32.1 (9.0); worse-ear PTA4 36.7 (9.6)
Wu et al. 2025 [16]	82	83	68.5 (7.1)	66.9 (6.6)	39 (47.6%)	42 (50.6%)	0	0	82 (100%)	83 (100%)	PTA 0.5/1/2/4 kHz: 35.6 (5.7), calculated	PTA 0.5/1/2/4 kHz: 36.5 (7.1), calculated
Yellamsetty and Lewis 2024 [15]	40	40	59.33 (12.3)	59.33 (12.3)	21 (52.5%)	21 (52.5%)	36 (90.0%)	36 (90.0%)	4 (10.0%)	4 (10.0%)	41.45 (10.89), 4-frequency PTA per ear	41.45 (10.89), 4-frequency PTA per ear
Zheng et al. 2025 [22]	32	32	58.0 (13.7)	62.0 (11.2)	15 (46.9%)	13 (40.6%)	20 (62.5%)	21 (65.6%)	12 (37.5%)	11 (34.4%)	PTA 0.5/1/2/4 kHz: 32.1 (9.0)	PTA 0.5/1/2/4 kHz: 35.1 (8.7)

kHz, kilohertz; N, number; NR, not reported; PTA, pure-tone average; PTA4, four-frequency pure-tone average; SD, standard deviation.

**Table 3:** GRADE evidence profile.

Outcomes	Participants (Studies)	Risk of Bias	Inconsistency	Indirectness	Imprecision	Publication Bias	Overall Certainty of Evidence	Effect Estimates
Patient-reported hearing benefit	1002 (6 RCTs)	serious ^a^	very serious ^b^	not serious	serious ^d^	not assessed ^f^	⊕◯◯◯ Very low	SMD 0.01 higher (0.44 lower to 0.46 higher)
Speech-in-noise/speech recognition	354 (4 RCTs)	serious ^a^	serious ^c^	not serious	serious ^d^	not assessed ^f^	⊕◯◯◯ Very low	SMD 0.11 higher (0.25 lower to 0.47 higher)
Hearing loss	734 (3 RCTs)	serious ^a^	serious ^c^	not serious	serious ^d^	not assessed ^f^	⊕◯◯◯ Very low	SMD 0.22 lower (0.50 lower to 0.06 higher)
Hearing-aid satisfaction	333 (3 RCTs)	serious ^a^	very serious ^b^	not serious	serious ^d^	not assessed ^f^	⊕◯◯◯ Very low	SMD 0.33 lower (0.96 lower to 0.30 higher)
Device use/adherence	700 (3 RCTs)	serious ^a^	very serious ^b^	not serious	serious ^d^	not assessed ^f^	⊕◯◯◯ Very low	SMD 0.12 lower (0.62 lower to 0.39 higher)
Need for follow-up adjustment	333 (3 RCTs)	serious ^a^	very serious ^b^	not serious	serious ^d^	not assessed ^f^	⊕◯◯◯ Very low	RR 0.46 (0.12 to 1.71)
Any adverse event	955 (4 RCTs)	serious ^a^	not serious	not serious	very serious ^e^	not assessed ^f^	⊕◯◯◯ Very low	RR 0.82 (0.21 to 3.17)

RCT: randomized controlled trial; RR: risk ratio; SMD: standardized mean difference. For crossover trials, participants were counted once in the participants column. **Explanations**: ^a^. Downgraded for risk of bias because several contributing trials had some concerns or a high overall risk of bias, mainly related to missing outcome data, outcome measurement, deviations from intended interventions, or selection of the reported result. ^b^. Downgraded two levels for very substantial heterogeneity (I^2^ > 75%) with variability in the direction and magnitude of study effects. ^c^. Downgraded for substantial heterogeneity (I^2^ between 50% and 75%) with clinically and statistically variable study effects. ^d^. Downgraded for imprecision because the confidence interval crossed the null and included a potentially important benefit and/or harm. ^e^. Downgraded two levels for imprecision because events were rare and the confidence interval was very wide, including both appreciable benefit and harm. ^f^. Publication bias was not assessed because fewer than 10 studies contributed to each outcome.

## Data Availability

All data are available within the manuscript and can be obtained from the corresponding authors upon a reasonable request.

## Supplementary Materials

The following supporting information can be downloaded at https://www.mdpi.com/article/10.3390/audiolres16050139/s1. Table S1: Search strategy; Table S2: Excluded records in full-text screening; Table S3: Characteristics of the OTC/self-fitting interventions; Table S4: Characteristics of the audiologist-fitted/conventional-care comparators. Figure S1: Leave-one-out sensitivity analysis for patient-reported hearing benefit; Figure S2: Galbraith plot for patient-reported hearing benefit; Figure S3: Leave-one-out sensitivity analysis for speech-in-noise/speech recognition; Figure S4: Galbraith plot for speech-in-noise/speech recognition; Figure S5: Leave-one-out sensitivity analysis for hearing handicap; Figure S6: Galbraith plot for hearing handicap; Figure S7: Leave-one-out sensitivity analysis for hearing-aid satisfaction; Figure S8: Galbraith plot for hearing-aid satisfaction; Figure S9: Leave-one-out sensitivity analysis for device use/adherence. Figure S10: Galbraith plot for device use/adherence; Figure S11: Leave-one-out sensitivity analysis for need for follow-up adjustment; Figure S12: Galbraith plot for need for follow-up adjustment.
