# Supplementary material for: Self-Fitting Versus Professional-Fitting Hearing Aids: A Systematic Review and Meta-Analysis of Randomized Controlled Trials
Source: Audiol Res. 2026 Sep 19;16(5):139. doi: 10.3390/audiolres16050139 (PMC13600142; doi:10.3390/audiolres16050139)
Supplement: Supplementary file 1 [file audiolres-16-00139-s001.zip › audiolres-4477414-supplementary.pdf]

Supplementary Materials:

Contents:

Tables.

Table S1. Search strategy.

Table S2. Excluded records in full-text screening.

Table S3. Characteristics of the OTC/self-fitting interventions.

Table S4. Characteristics of the audiologist-fitted/conventional-care comparators.

Figures.

Figure S1. Leave-one-out sensitivity analysis for patient-reported hearing benefit.

Figure S2. Galbraith plot for patient-reported hearing benefit.

Figure S3. Leave-one-out sensitivity analysis for speech-in-noise/speech recognition.

Figure S4. Galbraith plot for speech-in-noise/speech recognition.

Figure S5. Leave-one-out sensitivity analysis for hearing handicap.

Figure S6. Galbraith plot for hearing handicap.

Figure S7. Leave-one-out sensitivity analysis for hearing-aid satisfaction.

Figure S8. Galbraith plot for hearing-aid satisfaction.

Figure S9. Leave-one-out sensitivity analysis for device use/adherence.

Figure S10. Galbraith plot for device use/adherence.

Figure S11. Leave-one-out sensitivity analysis for need for follow-up adjustment.

Figure S12. Galbraith plot for need for follow-up adjustment.

Table S1: Search Strategy.

Database	Search Terms	Search Field	Search Results
PubMed	(("Hearing Aids"[Mesh] OR "hearing aid"[tiab] OR "hearing aids"[tiab] OR "hearing device"[tiab] OR "hearing devices"[tiab] OR "amplification device"[tiab] OR "amplification devices"[tiab]) AND ("over-the-counter"[tiab] OR OTC[tiab] OR "self-fit"[tiab] OR "self-fitted"[tiab] OR "self-fitting"[tiab] OR "self fitting"[tiab] OR "self-adjusted"[tiab] OR "self-adjustable"[tiab] OR "self-selected"[tiab] OR "self-programmed"[tiab] OR "self-programming"[tiab] OR "person-fit"[tiab] OR "person-fitted"[tiab] OR "person fitting"[tiab] OR "user-fit"[tiab] OR "user-fitted"[tiab] OR "user-adjusted"[tiab] OR "user-operated"[tiab] OR "consumer-fit"[tiab] OR "consumer-fitted"[tiab] OR "direct-to-consumer"[tiab] OR "app-guided"[tiab] OR "app based"[tiab] OR "app-based"[tiab] OR "smartphone-based"[tiab] OR "preset"[tiab] OR "presets"[tiab]))	MeSH Terms; Title/Abstract	311
Cochrane CENTRAL	([mh "Hearing Aids"] OR "hearing aid",ab,kw OR "hearing aids",ab,kw OR "hearing device",ab,kw OR "hearing devices",ab,kw OR "amplification device",ab,kw OR "amplification devices",ab,kw) AND ("over-the-counter",ab,kw OR OTC,ab,kw OR "self-fit",ab,kw OR "self-fitted",ab,kw OR "self-fitting",ab,kw OR "self fitting",ab,kw OR "self-adjusted",ab,kw OR "self-adjustable",ab,kw OR "self-selected",ab,kw OR "self-programmed",ab,kw OR "self-programming",ab,kw OR "person-fit",ab,kw OR "person-fitted",ab,kw OR "person fitting",ab,kw OR "user-fit",ab,kw OR "user-fitted",ab,kw OR "user-adjusted",ab,kw OR "user-operated",ab,kw OR "consumer-fit",ab,kw OR "consumer-fitted",ab,kw OR "direct-to-consumer",ab,kw OR "app-guided",ab,kw OR "app based",ab,kw OR "app-based",ab,kw OR "smartphone-based",ab,kw OR preset,ab,kw OR presets,ab,kw)	MeSH descriptor; Title, Abstract, Keyword	65
Web of Science	TS=(("hearing aid" OR "hearing aids" OR "hearing device" OR "hearing devices" OR "amplification device" OR "amplification devices") AND ("over-the-counter" OR OTC OR "self-fit" OR "self-fitted" OR "self-fitting" OR "self fitting" OR "self-adjusted" OR "self-adjustable" OR "self-selected" OR "self-programmed" OR "self-programming" OR "person-fit" OR "person-fitted" OR "person fitting" OR "user-fit" OR "user-fitted" OR "user-adjusted" OR "user-operated" OR "consumer-fit" OR "consumer-fitted" OR "direct-to-consumer" OR "app-guided" OR "app based" OR "app-based" OR "smartphone-based" OR preset OR presets))	Topic	271
Scopus	TITLE-ABS-KEY(("hearing aid" OR "hearing aids" OR "hearing device" OR "hearing devices" OR "amplification device" OR "amplification devices") AND ("over-the-counter" OR OTC OR "self-fit" OR "self-fitted" OR "self-fitting" OR "self fitting" OR "self-adjusted" OR "self-adjustable" OR "self-selected" OR "self-programmed" OR "self-programming" OR "person-fit" OR "person-fitted" OR "person fitting" OR "user-fit" OR "user-fitted" OR "user-adjusted" OR "user-operated" OR "consumer-fit" OR "consumer-fitted" OR "direct-to-consumer" OR "app-guided" OR "app based" OR "app-based" OR "smartphone-based" OR preset OR presets))	Title, Abstract, Keywords	396
Embase	(exp hearing aid/ OR "hearing aid".ti,ab,kw. OR "hearing aids".ti,ab,kw. OR "hearing device".ti,ab,kw. OR "hearing devices".ti,ab,kw. OR "amplification device".ti,ab,kw. OR "amplification devices".ti,ab,kw.) AND ("over-the-counter".ti,ab,kw. OR OTC.ti,ab,kw. OR "self-fit".ti,ab,kw. OR "self-fitted".ti,ab,kw. OR "self-fitting".ti,ab,kw. OR "self fitting".ti,ab,kw. OR "self-adjusted".ti,ab,kw. OR "self-adjustable".ti,ab,kw. OR "self-	Emtree; Title, Abstract, Keywords	441

	selected".ti,ab,kw. OR "self-programmed".ti,ab,kw. OR "self-programming".ti,ab,kw. OR "person-fit".ti,ab,kw. OR "person-fitted".ti,ab,kw. OR "person fitting".ti,ab,kw. OR "user-fit".ti,ab,kw. OR "user-fitted".ti,ab,kw. OR "user-adjusted".ti,ab,kw. OR "user-operated".ti,ab,kw. OR "consumer-fit".ti,ab,kw. OR "consumer-fitted".ti,ab,kw. OR "direct-to-consumer".ti,ab,kw. OR "app-guided".ti,ab,kw. OR "app based".ti,ab,kw. OR "app-based".ti,ab,kw. OR "smartphone-based".ti,ab,kw. OR preset.ti,ab,kw. OR presets.ti,ab,kw.)		

Table S2: Excluded records in full-text screening.

Title	Published Year	DOI	Study ID	Exclusion reason
Usability of Over-the-Counter Hearing Aids in Older Adults.	2025	10.1044/2025_AJA-25-00013	Coco 2025	Wrong study design
Over-the-Counter Hearing Aids Versus Traditional Hearing Aids in Patients with Mild-to-Moderate Hearing Loss: Protocol for a Noninferiority Randomized Controlled Trial.	2024	10.2196/59894	Kim 2024	Study protocol
Comparing Self-Fitting Strategies for Over-the-Counter Hearing Aids: A Crossover Clinical Trial.	2024	10.1001/jamaoto.2024.2007	Knoetze 2024	Wrong comparator
Validation of a Self-Fitting Method for Over-the-Counter Hearing Aids.	2020	10.1177/2331216519900589	Sabin 2020	Wrong study design
Outcomes With a Self-Fitting Hearing Aid.	2018	10.1177/2331216518768958	Keidser 2018	Wrong study design
Over-the-counter hearing aids reduce depression symptoms in older adults with hearing loss: a randomized controlled trial	2025	10.1038/s44220-025-00408-4	Jiang 2025	Wrong intervention
Effectiveness of an over-the-counter self-fitting hearing aid compared to an audiologist-fitted hearing aid: A randomized clinical trial	2022	10.1101/2022.12.12.22283331	DeSousa 2022	A preprint of an already included trial
Comparison of Hearing Aid Fitting Outcomes Between Self-fit and Professional Fit for MDHearing Smart Hearing Aids	2021	NCT05165121	NCT05165121	Study protocol
Clinical Validation of the Lexie Lumen Hearing Aid	2022	NCT05337748,	NCT05337748 2022	Study protocol
Validating a Self-fitting Hearing Aid	2022	NCT05246904,	NCT05246904 2022	Study protocol
Innovations in Hearing Accessibility and Technology (IHAT) Study	2024	NCT06689865,	NCT06689865 2024	Study protocol
Wear-Time Trial for Self-Fitting Hearing Aid	2021	NCT04823494,	NCT04823494 2021	Study protocol
Clinical Investigation and Validation of a Self-fitted Air-conduction Hearing Aid	2023	NCT06167161,	NCT06167161 2023	Study protocol
OTC HA vs HA in patients with mild-to-moderate HL	2023	KCT0008666,	KCT0008666 2023	Study protocol

Table S3: Characteristics of the OTC/self-fitting interventions.

Study ID	OTC / Self-fitting Label	Intervention device/model	Device category	Selection pathway	Fitting/programming pathway	Professional involvement	Real-ear verification	Support/training	Home-use duration
Baltzell et al. 2025	Self-fitted	Concha Sol	FDA-approved self-fitting OTC, RIC digital HA with 16 WDRC channels	Audiologist selected receiver length and open-dome size once; participant then completed self-fitting through Concha app/Soundscope	Soundscope generated ear-specific gain profiles through in-app audibility and paired-preference comparisons; volume rocker available during field use	Audiologist paired devices/opened app, then left booth; no active fitting guidance during self-fit	No	Minimal; app-guided self-fitting; volume adjustment only during field use	2-week field-use period
De Sousa et al. 2023	Self-fitting (SF)	Lexie Lumen	FDA-approved self-fitting OTC BTE digital HA	Participants received commercial packaging and used supplied instructions/app independently	Lexie app used in-situ thresholds at 05–6 kHz and proprietary self-fitting algorithm; no support/fine-tuning allowed for first 2 weeks	None during initial fitting; remote support became available only after 2-week assessment	No	No support first 2 weeks; after T1, remote Lexie support/fine-tuning available on request; 2 SF participants used it	2-week initial trial, followed by a 4-week trial (6 weeks total)
De Sousa et al. 2024	Self-fit	Lexie Lumen	FDA-approved self-fitting OTC BTE digital HA	Same original parent-trial allocation; participants retained devices after the 6-week trial	Same self-fit pathway as De Sousa 2023; no new fitting intervention at extension	No new professional refitting reported; post-trial queries directed through remote support	No	Remote support channel available after parent trial; only rare support requests reported	Approximately 8 months
Humes et al. 2017	Consumer decides (CD) / OTC	ReSound Alera 9 mini-behind-the-ear (BTE)	Conventional high-end digital mini-BTE devices delivered via simulated OTC consumer-decides model, not an FDA OTC-labeled device	Participants self-selected preprogrammed devices; color and preconfigured gain/program options were available	Devices were preprogrammed; CD model used consumer selection rather than individualized audiologist programming	Minimal/no audiologist fitting in CD arm; AB comparator had audiologist-based best-practice fitting and orientation	No	CD participants received written user guide; AB comparator received 45–60-min orientation/counseling	6-week trial
Humes et al. 2025	Self-fit/person-fit methods: CD and EF	GN ReSound LiNX Quattro 9 rechargeable receiver-in-the-canal hearing aids	Conventional rechargeable RIC/BTE HAs used to test preset/self-fit OTC-like methods	Participants selected receiver wire length, dome size, and color; then ranked four preprogrammed responses using a device-fitting app after listening to speech/music/environmental sounds	CD varied overall gain; EF varied high-frequency gain; devices were not individually programmed to each participant’s audiogram; preferred program became default	Audiologist retrieved preassembled devices and reordered programs according to participant selection; no use/care instruction beyond written materials	No	Written user/troubleshooting guides only; study contact available but responses referred participants back to written materials; no phone/app connectivity allowed during study	6 weeks and 6 months
Wu et al. 2025	OTC	High-end and low-end behind-the-ear prescription hearing aid models	Simulated preset-based OTC HAs using prescription devices; not commercial OTC devices	OTC participants self-selected 1 of 4 gain-frequency presets using a tablet kiosk app; OTC+ involved audiologist-assisted preset/dome selection	Four predetermined gain-frequency responses; participants selected preferred preset; OTC+ allowed limited app-based adjustment by audiologist	OTC: no audiologist support for selection	No	OTC: manuals/quick guides/simulated websites; could request preset reselection/replacement	6 to 7 weeks

Yellamsetty & Lewis 2024	SELF-FIT	Receiver-in-canal (RIC) hearing aids with single vent domes	OTC self-fitting RIC hearing aids	Participants completed both SELF-FIT and BP-FIT conditions in randomized crossover order; same physical RIC/single-vent-dome setup	SELF-FIT settings generated by user-directed self-fitting software/algorithm	Professional involvement for study testing and reprogramming between conditions; self-fit condition itself used self-fitting software	Evaluated and measured objectively via REM for comparison, but REM was not used to adjust the SELF-FIT programming	Field use without washout; subjective APHAB after each 2-week condition	2 weeks
Zheng et al. 2025	Self-fit	LifeEar CORE	FDA-approved BTE digital OTC HA with volume/tone controls and four programs	Participants received preassembled LifeEar CORE devices; clinician could assist dome/tubing choice if needed to minimize physical-fit effects	Participants followed user manual, downloaded app, paired HAs, completed in-app thresholds at 500–6000 Hz; app automatically programmed all four programs using NAL-NL2; users could adjust volume/tone/programs	Limited to physical-fit help when needed and app-operation demonstration; pro-fit comparator used audiology clinicians	No	User manual and app-guided setup; pro-fit group received counseling on HA use/management	1 month (30 days)

AB, audiologist-based best-practice fitting; APHAB, Abbreviated Profile of Hearing Aid Benefit; BP-FIT, best-practice fit; BTE, behind-the-ear; CD, consumer-decides; dB, decibel; EF, experimental self-fit/person-fit method with high-frequency gain variation; FDA, Food and Drug Administration; HA, hearing aid; HAs, hearing aids; Hz, hertz; kHz, kilohertz; NAL-NL2, National Acoustic Laboratories nonlinear fitting procedure, version 2; OTC, over-the-counter; OTC+, over-the-counter plus limited audiologist assistance; REM, real-ear measurement; RIC, receiver-in-canal; SELF-FIT, self-fitting condition; SF, self-fitting; T1, first follow-up time point; WDRC, wide dynamic range compression.

Table S4: Characteristics of the audiologist-fitted/conventional-care comparators.

Study ID	Audiologist-fitted / Conventional-Care Label	Audiologist-fitted / Conventional-Care Device	Audiologist-fitted / Conventional-Care Category	Audiologist-fitted / Conventional-Care pathway	Professional involvement	Real-ear verification	Same device across arms?
Baltzell et al. 2025	Clinician-fitted intervention	Concha Sol	FDA-approved RIC digital hearing aid used as clinician-fitted comparator	Research audiologist fitted devices bilaterally in sound booth using manufacturer fitting software; Comfort Logix fitting rationale; static directional microphone; manual WDRC adjustment only if immediate sound-quality complaints	Research audiologist performed the fitting; no REM or NAL-NL2 because NAL-NL2 was unavailable in the software	No	Yes
De Sousa et al. 2023	Audiologist-fitted group / audiologist-fitted best practices	Lexie Lumen	FDA-approved self-fitting OTC BTE digital hearing aid, fitted by audiologist in comparator arm	Audiologists fitted the same hearing aid using calibrated audiometric thresholds, NAL-NL2 prescriptive gain targets, real-ear verification within 5 dB tolerance, and hearing-aid use instruction; fine-tuning available after 2 weeks on request	Registered research audiologists performed fitting, verification, instruction, and requested fine-tuning	Yes	Yes
De Sousa et al. 2024	Audiologist-fit device group	Lexie Lumen	Same FDA-approved OTC device from parent RCT, followed long term	No new fitting intervention; participants were followed after the original audiologist-fit pathway from De Sousa 2023, which used best practices including REM, fine-tuning, and orientation	Original fitting by audiologists; post-trial support queries directed through remote support channel	Yes	Yes
Humes et al. 2017	Audiology best practices (AB)	ReSound Alera 9	Conventional high-end digital mini-BTE hearing aids	Audiologist selected dome/tubing, programmed devices to NAL-NL2 targets using ReSound Aventa software, verified/fine-tuned output with probe-tube/real-ear measures, checked maximum output, and provided structured orientation	Multiple audiologists: one/two for programming/REM/fine-tuning and another blinded audiologist for 45–60-min orientation	Yes	Yes
Humes et al. 2025	Audiologist-based best-practices fitting method (AB)	GN ReSound LiNX Quattro 9	Conventional rechargeable RIC/BTE hearing aids used in best-practice arm	Audiologist selected receiver wire/dome/color, programmed using audiogram and manufacturer software to NAL-NL2 targets, verified 65-dB ISTS real-ear gain, collected 55/65/75-dB aided responses, checked maximum output, and provided standardized orientation/counseling	Audiologist performed physical fitting, programming, REM verification/fine-tuning, maximum-output checks, and orientation	Yes	Yes
Wu et al. 2025	Audiologist service model (AUD)	High-end and low-end HA models (models were not named explicitly)	Prescription hearing aids; high-end and low-end technology levels	Audiologists fitted prescription HAs following best-practice guidelines: audiometry, COSI, loudness discomfort levels, unaided QuickSIN, NAL prescription, probe-microphone/real-ear verification, orientation, counseling, mandatory 1-week follow-up, and additional visits as needed	Licensed audiologists delivered AUD services; OTC+ involved limited audiologist services; OTC involved independent use	Yes	Yes
Yellamsetty & Lewis 2024	Best Practice Fit (BP-FIT)	Receiver-in-canal (RIC) hearing aids	OTC/self-fitting hearing-aid system used in professionally fitted mode	Professionally trained audiologist performed BP-FIT; settings based on professional audiometric thresholds and adjusted to NAL-NL2 targets; participants crossed over between BP-FIT and SELF-FIT	Audiologist conducted BP-FIT and reprogramming between conditions	Yes	Yes
Zheng et al. 2025	Professional-fit / pro-fit group	LifeEar CORE	FDA-approved BTE digital OTC hearing aid fitted professionally	Audiology clinicians fitted the same OTC devices using audiometer thresholds; measured real-ear-to-coupler difference; used fitting portal/app to meet NAL-NL2 targets for 55/65/75 dB SPL inputs on Verifit2; adjusted gain based on descriptive targets and counseled participants	Audiology clinicians performed fitting, target matching, gain adjustment, and counseling	Yes	Yes

AB, audiology-based best-practices; AUD, audiologist service model; BP-FIT, best-practice fit; BTE, behind-the-ear; COSI, Client Oriented Scale of Improvement; dB, decibel; FDA, Food and Drug Administration; HA, hearing aid; NAL, National Acoustic Laboratories; NAL-NL2, National Acoustic Laboratories nonlinear fitting procedure, version 2; OTC, over-the-counter; OTC+, over-the-counter plus limited audiologist assistance; QuickSIN, Quick Speech-in-Noise test; RCT, randomized controlled trial; REM, real-ear measurement; RIC, receiver-in-canal; SELF-FIT, self-fitting condition; WDRC, wide dynamic range compression.

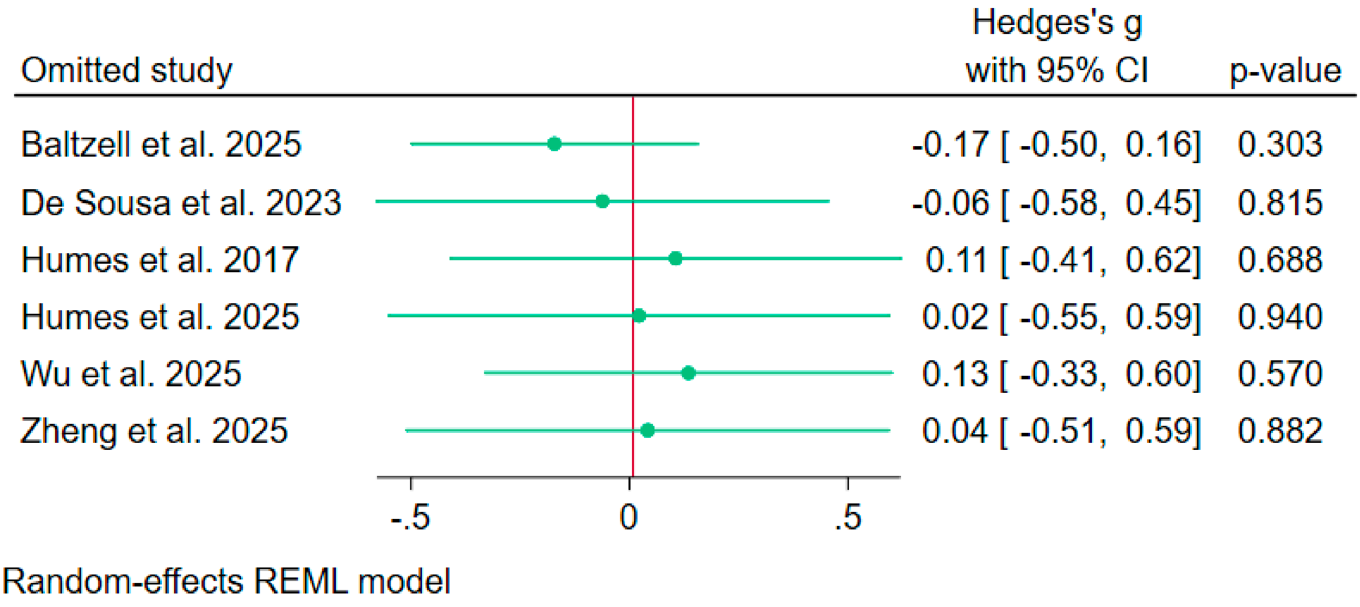

Figure S1: Leave-one-out sensitivity analysis for patient-reported hearing benefit.

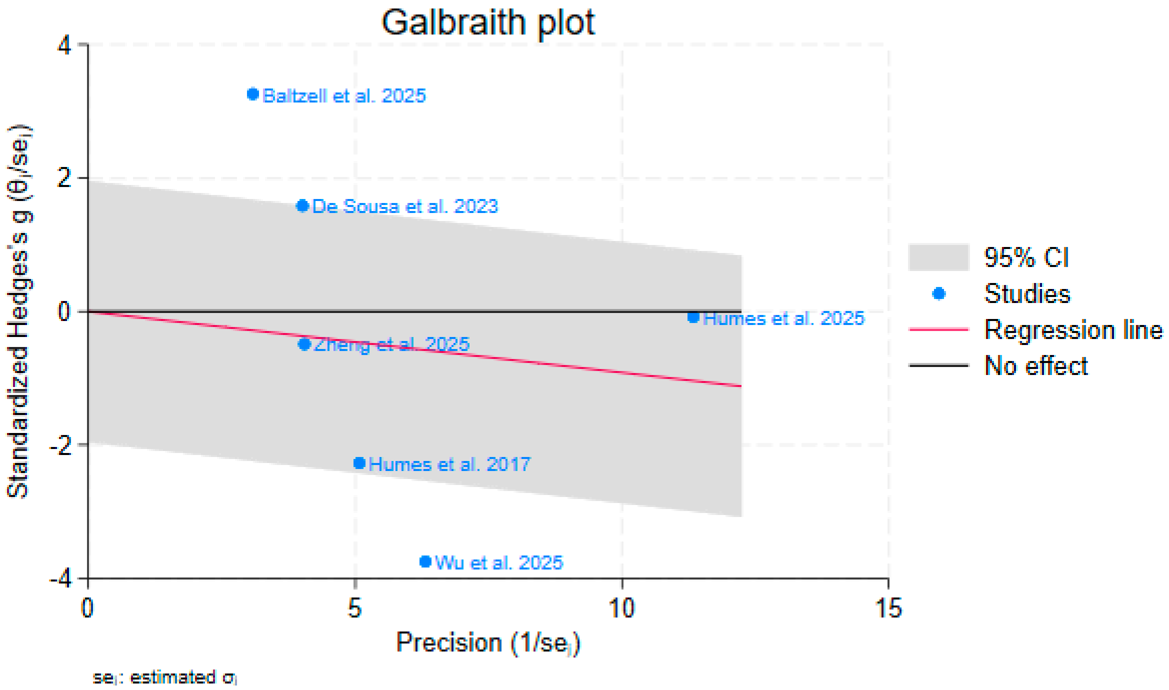

Figure S2: Galbraith plot for patient-reported hearing benefit.

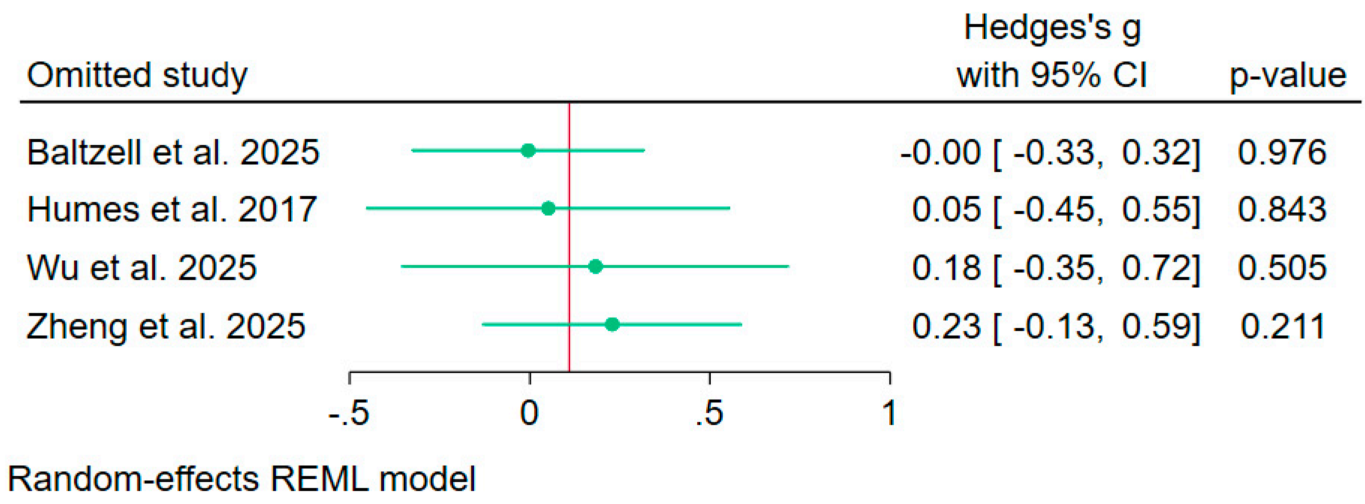

Figure S3: Leave-one-out sensitivity analysis for speech-in-noise/speech recognition.

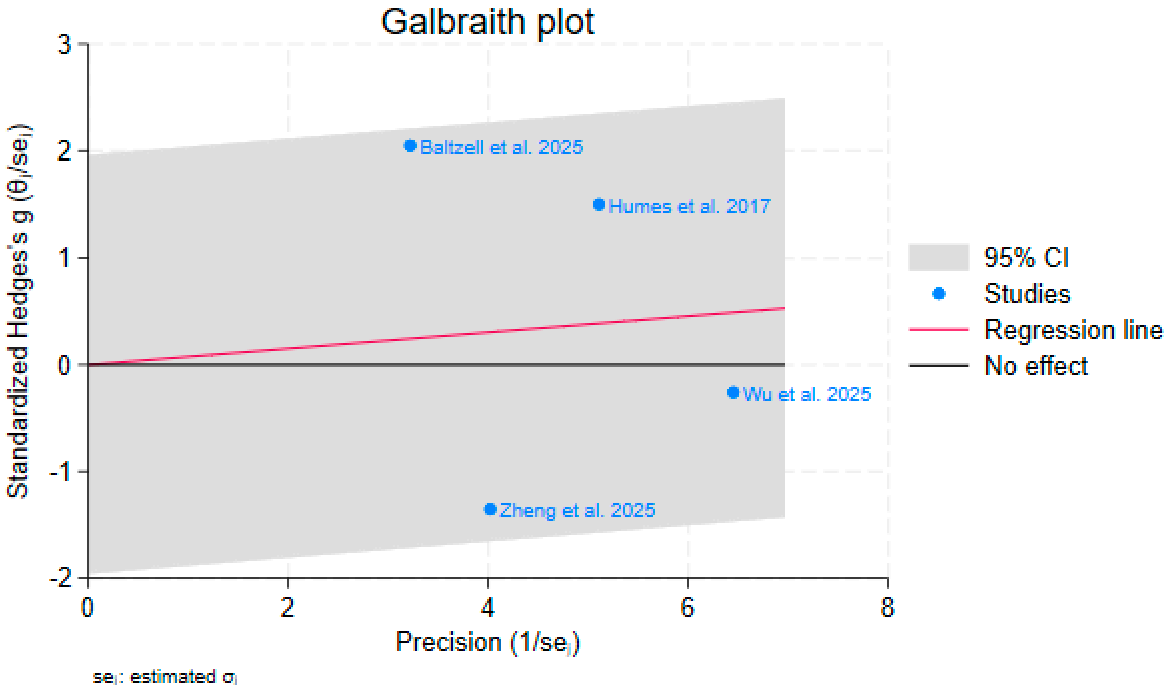

Figure S4: Galbraith plot for speech-in-noise/speech recognition.

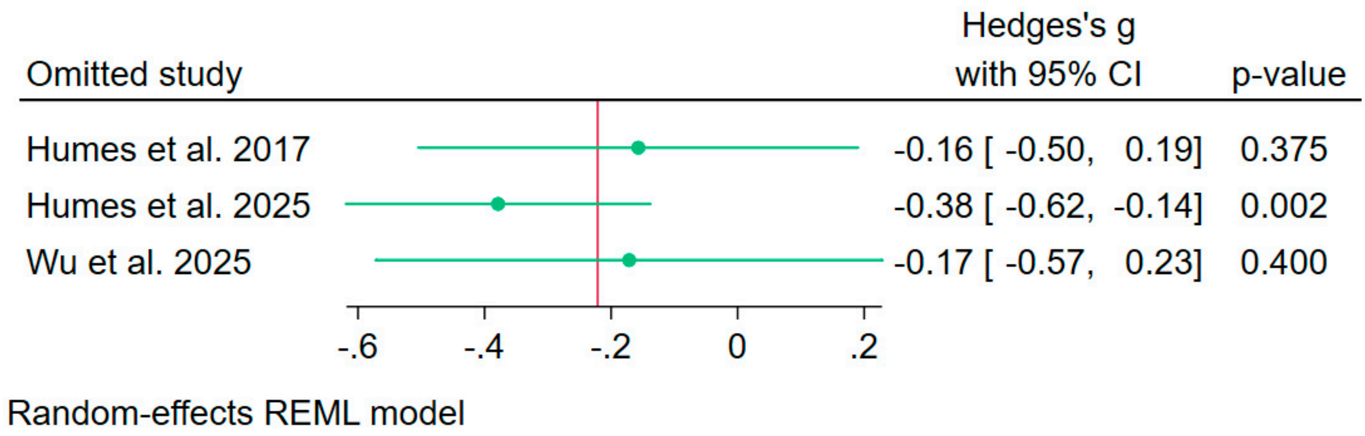

Figure S5: Leave-one-out sensitivity analysis for hearing handicap.

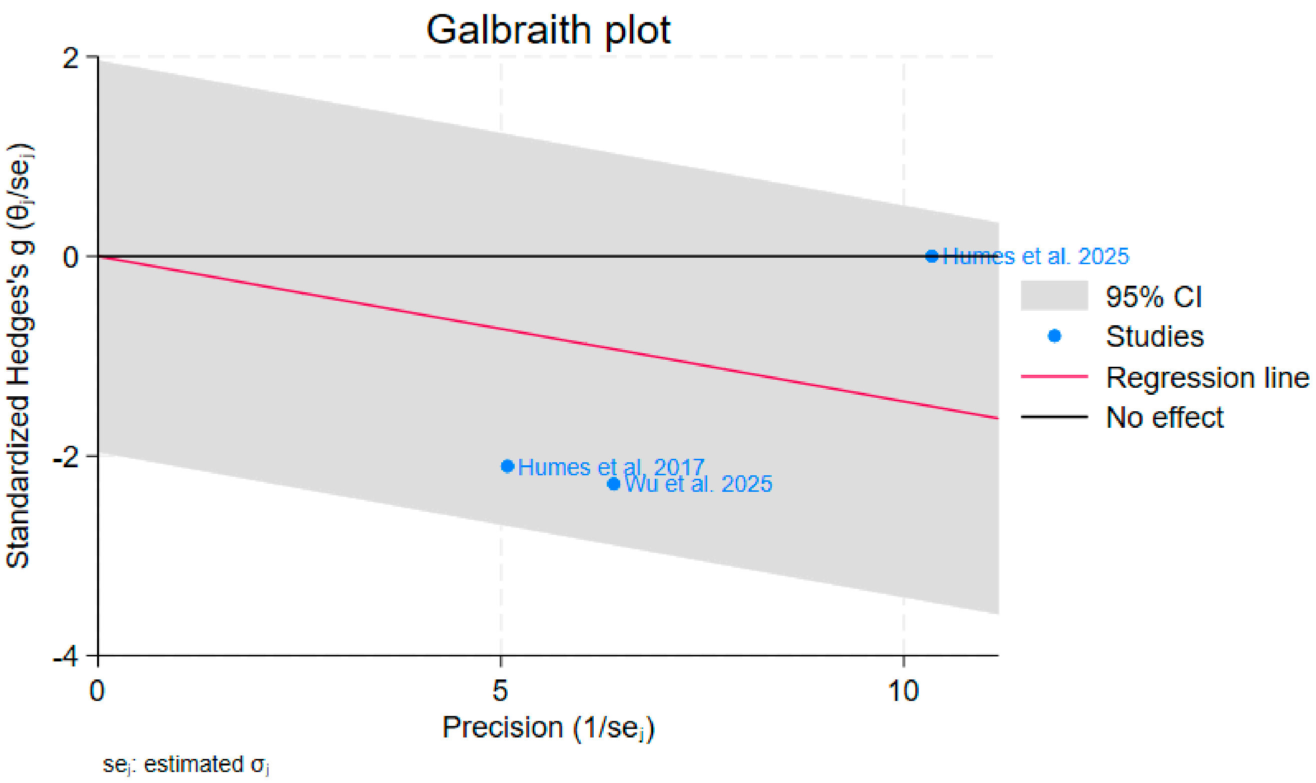

Figure S6: Galbraith plot for hearing handicap.

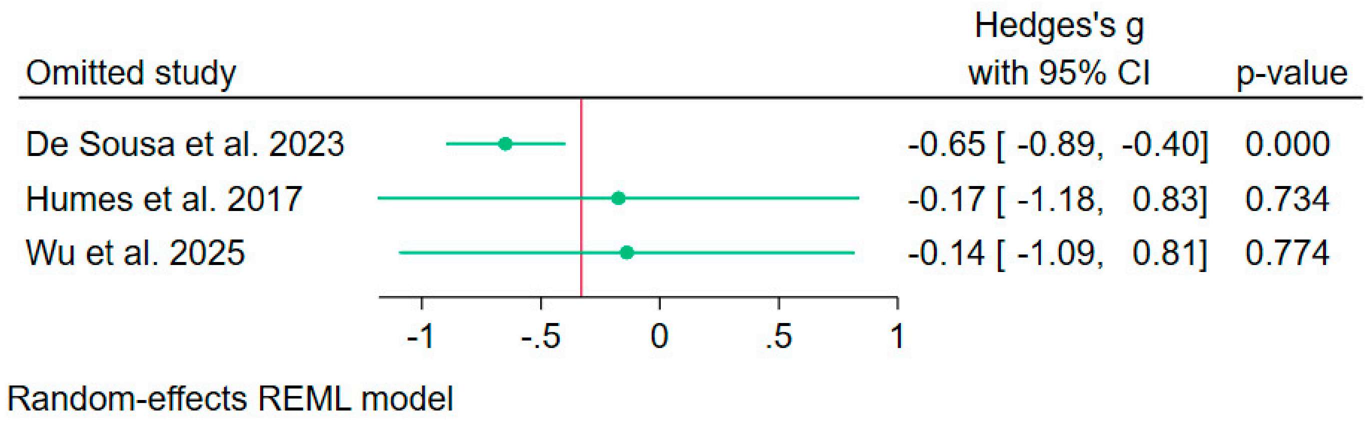

Figure S7: Leave-one-out sensitivity analysis for hearing-aid satisfaction.

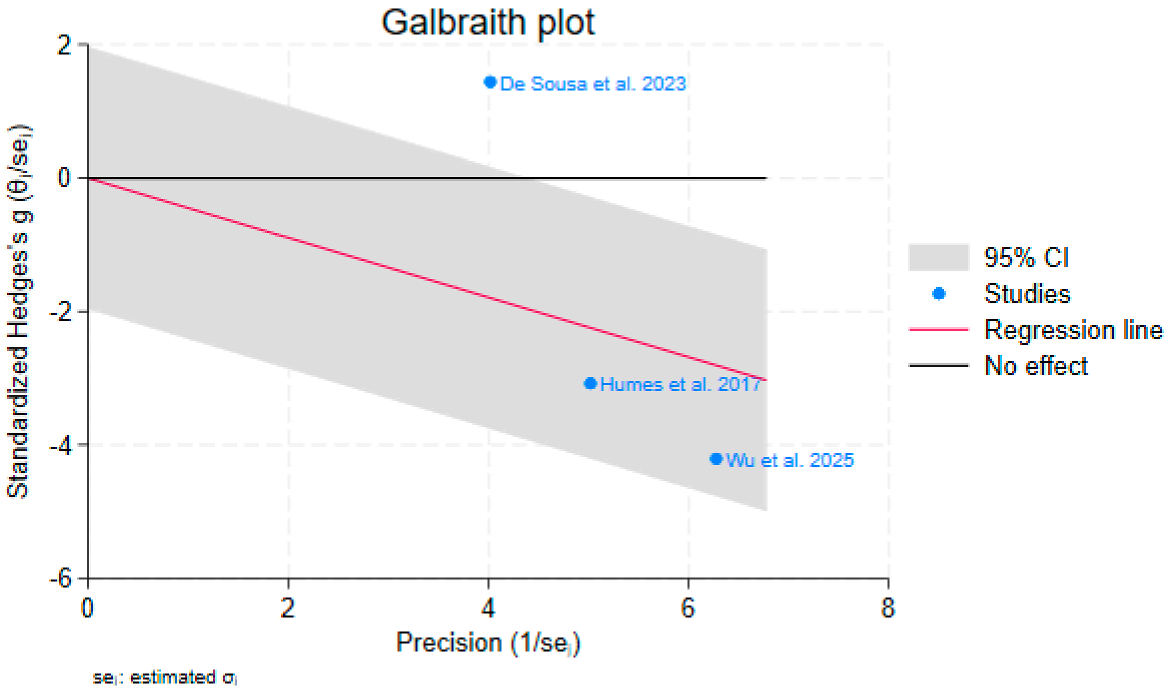

Figure S8: Galbraith plot for hearing-aid satisfaction.

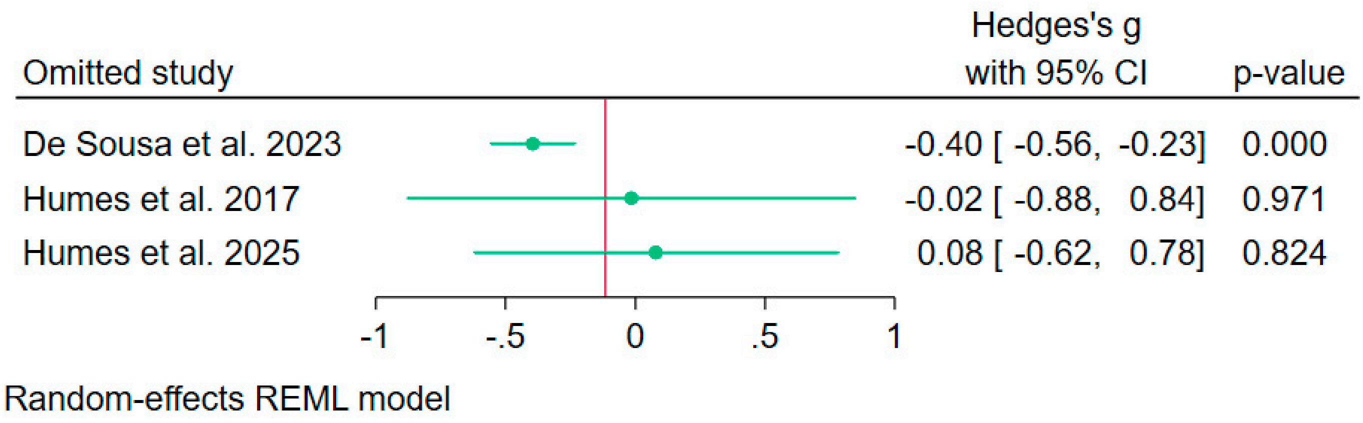

Figure S9: Leave-one-out sensitivity analysis for device use/adherence.

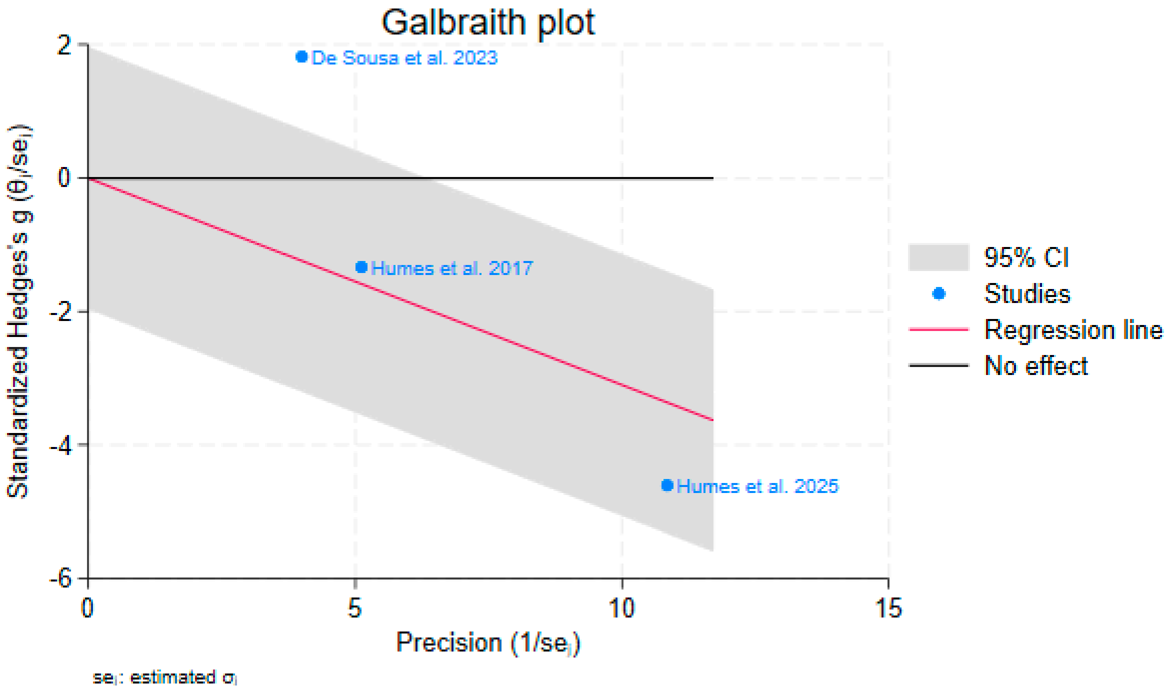

Figure S10: Galbraith plot for device use/adherence.

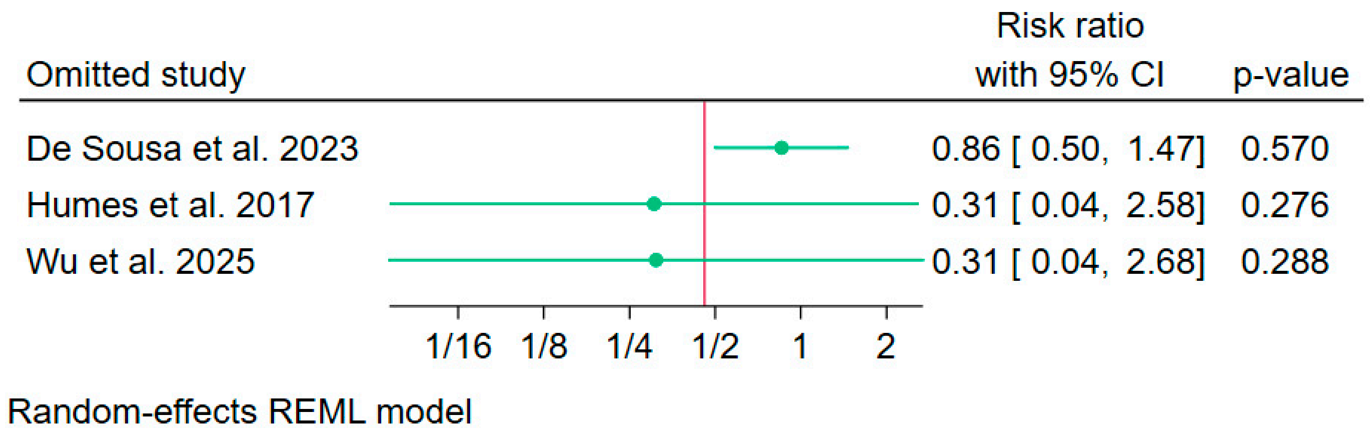

Figure S11: Leave-one-out sensitivity analysis for need for follow-up adjustment.

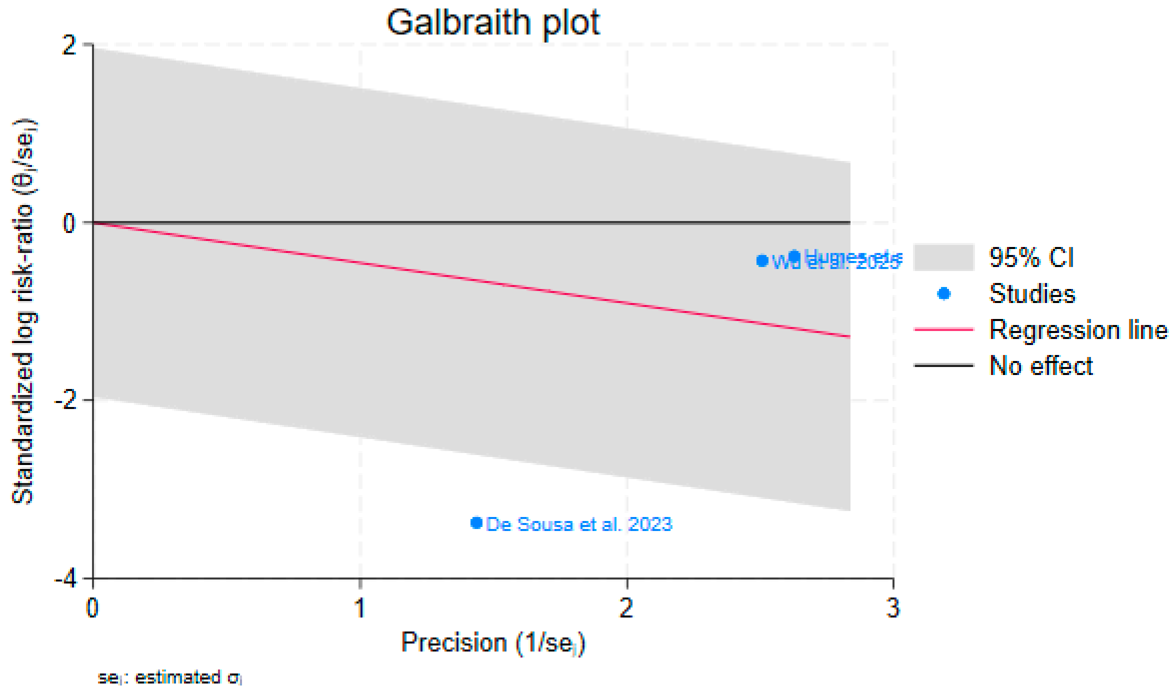

Figure S12: Galbraith plot for need for follow-up adjustment.
